# N-WASP is required for Amphiphysin-2/BIN1-dependent nuclear positioning and triad organization in skeletal muscle and is involved in the pathophysiology of centronuclear myopathy

**DOI:** 10.15252/emmm.201404436

**Published:** 2014-09-29

**Authors:** Sestina Falcone, William Roman, Karim Hnia, Vincent Gache, Nathalie Didier, Jeanne Lainé, Frederic Auradé, Isabelle Marty, Ichizo Nishino, Nicolas Charlet-Berguerand, Norma Beatriz Romero, Giovanna Marazzi, David Sassoon, Jocelyn Laporte, Edgar R Gomes

**Affiliations:** 1 Myology Group, UMR S 787 INSERM, Université Pierre et Marie Curie Paris 6Paris, France; 2 Institut de Myologie, Groupe Hospitalier Pitié-SalpêtrièreParis, France; 3 IGBMC-CNRS, UMR 7104 INSERM, U964Illkirch, France; 4 Morphology Unit, Myology Institut, Pitié Salpêtrière HospitalParis, France; 5 INSERM U836, Grenoble Institut des Neurosciences, Equipe Muscle et PathologiesGrenoble, France; 6 National Center of Neurology and PsychiatryTokyo, Japan; 7 Instituto de Medicina Molecular, Faculdade de Medicina da Universidade de LisboaLisboa, Portugal

**Keywords:** centronuclear myopathy, cytoskeleton, nuclear movement, triad formation

## Abstract

Mutations in amphiphysin-2/BIN1, dynamin 2, and myotubularin are associated with centronuclear myopathy (CNM), a muscle disorder characterized by myofibers with atypical central nuclear positioning and abnormal triads. Mis-splicing of amphiphysin-2/BIN1 is also associated with myotonic dystrophy that shares histopathological hallmarks with CNM. How amphiphysin-2 orchestrates nuclear positioning and triad organization and how CNM-associated mutations lead to muscle dysfunction remains elusive. We find that N-WASP interacts with amphiphysin-2 in myofibers and that this interaction and N-WASP distribution are disrupted by amphiphysin-2 CNM mutations. We establish that N-WASP functions downstream of amphiphysin-2 to drive peripheral nuclear positioning and triad organization during myofiber formation. Peripheral nuclear positioning requires microtubule/Map7/Kif5b-dependent distribution of nuclei along the myofiber and is driven by actin and nesprins. In adult myofibers, N-WASP and amphiphysin-2 are only involved in the maintenance of triad organization but not in the maintenance of peripheral nuclear positioning. Importantly, we confirmed that N-WASP distribution is disrupted in CNM and myotonic dystrophy patients. Our results support a role for N-WASP in amphiphysin-2-dependent nuclear positioning and triad organization and in CNM and myotonic dystrophy pathophysiology.

## Introduction

Centronuclear myopathy (CNM) is a rare neuromuscular disease associated with skeletal muscle weakness and hypotonia (Pierson *et al*, 2005; Nicot *et al*, 2007). CNM muscle fibers from patients and mouse models have centrally positioned nuclei and exhibit defects in T-tubules, triads, and excitation–contraction coupling (Al-Qusairi *et al*, 2009; Dowling *et al*, 2009; Toussaint *et al*, 2011). Unlike many myopathies, centrally positioned nuclei in CNMs are not linked to excessive degeneration–regeneration processes. Several forms of CNM have been described in humans including the X-linked form (XLCNM, OMIM#310400), which exhibits the most severe phenotype and affects newborns, due to mutations in myotubularin (*MTM1*), a phosphoinositide phosphatase (Laporte *et al*, 1996), the autosomal dominant form (ADCNM, OMIM#160150) due to mutations in dynamin 2 (*DNM2*), a large GTPase (Bitoun *et al*, 2005), and an autosomal recessive form (ARCNM, OMIM#255200) due to mutations in *AMPH2/BIN1* (Nicot *et al*, 2007).

During skeletal muscle development, nuclei move toward the center of the myotube after myoblast fusion and spread along the center of the myotube prior to positioning at the periphery of the myofiber (Cadot *et al*, 2012; Metzger *et al*, 2012). Nuclear spreading along the myotube is mediated by microtubules, Map7, and Kif5b microtubule-binding proteins and is independent of nesprin proteins (Metzger *et al*, 2012). Nesprins are nuclear envelope proteins that tether the nucleus to the actin cytoskeleton (Zhang *et al*, 2007). Myofibers lacking nesprins exhibit peripheral nuclear positioning defects, but it is unknown at which stage of skeletal muscle development nesprins are involved in nuclear positioning. Furthermore, how CNM mutated genes are involved in nuclear positioning is also unknown.

Amphiphysin-2 (amph2), encoded by *AMPH2/BIN1*, is involved in T-tubule biogenesis and a skeletal muscle-specific isoform of amph2 expressing exon 11 is localized to the T-tubules (Butler *et al*, 1997; Lee *et al*, 2002; Toussaint *et al*, 2011). T-tubules are invaginations of the plasma membrane (sarcolemma) that become associated and transversally paired with a specific region of the sarcoplasmic reticulum (SR), the junctional SR/terminal cisternae. The structure formed between one T-tubule and two junctional SR compartments is named triad (Flucher *et al*, 1991). Triads couple the excitation of the sarcolemma and T-tubules with the release of calcium from the SR leading to muscle contraction, a process named excitation–contraction (EC) coupling. Multiple proteins are specifically localized in these structures, but the molecular mechanisms involved in triad organization are still poorly understood (Al-Qusairi & Laporte, 2011).

Alternative splicing of amph2 has been recently found in skeletal muscle biopsies from patients with myotonic dystrophy. CNM and myotonic dystrophy share some histopathological features such as centrally positioned nuclei and defects in T-tubules and triads (Fugier *et al*, 2011), suggesting a common unknown mechanism involving a role for amph2 on nuclear positioning and triad function in both CNM and myotonic dystrophy.

Amph2 has an N-terminal BAR domain responsible for membrane binding and curvature, and a C-terminal SH3 domain implicated in protein–protein interaction. These domains are mutated in ARCNM patients (Nicot *et al*, 2007; Prokic *et al*, 2014). SH3 domains interact and regulate multiple protein machineries, in particular different actin nucleation promoting factors involved in membrane remodeling (Suetsugu & Gautreau, 2012). N-WASP is an actin nucleation promoting factor that activates the Arp2/3 complex, an actin nucleator (Rohatgi *et al*, 1999). In skeletal muscle, N-WASP was recently found to be involved in myoblast fusion, nebulin-dependent actin nucleation during myofibrillogenesis, and IGF-1-dependent muscle hypertrophy (Takano *et al*, 2010; Gruenbaum-Cohen *et al*, 2012). However, a role for N-WASP in nuclear positioning and triad organization is unknown. Amphiphysin-1, a member of the amphiphysin family expressed primarily in the brain, regulates actin dynamics and membrane remodeling during endocytosis through activation of N-WASP (Yamada *et al*, 2009). Furthermore, a cardiac muscle-specific amph2 isoform interacts with N-WASP *in vitro* (Hong *et al*, 2014). We therefore explored the potential involvement of N-WASP downstream of amph2, a homolog of amphiphysin-1, in nuclear positioning and triad organization during skeletal myofiber maturation and its role in the pathophysiology of centronuclear myopathy and myotonic dystrophy.

## Results

### *In vitro* maturation of myofibers with peripheral nuclei and organized triads

**Figure 1:**
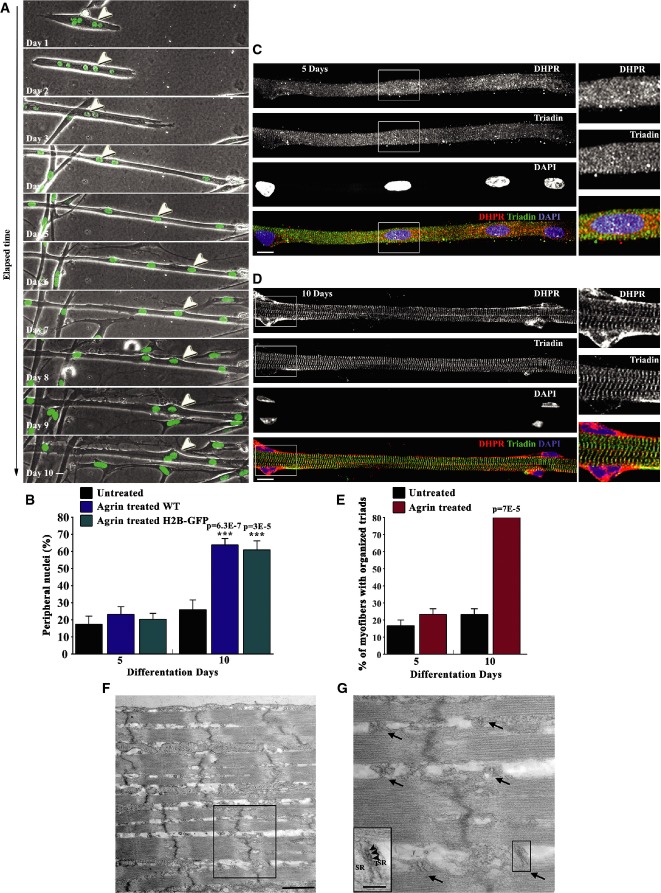
Peripheral localization of nuclei and organization of transversal T-tubules paired with sarcoplasmic reticulum (SR) in *in vitro* myofibers Images from a representative time-lapse dual color phase-contrast movie of H2B-GFP myotubes (Supplementary Movie S1), recorded from day 1 (after agrin addition) until day 10, showing nuclear positioning to the periphery during myofiber maturation. Arrowhead indicates an example of a nucleus going to the periphery. Bar, 15 µm.Quantification of peripheral nuclei in H2B-GFP and WT myofibers, treated or untreated with agrin and differentiated for 5 or 10 days. Error bars, s.e.m., *n* = 3. *P*-values from *t*-test (untreated versus treated condition).Representative immunofluorescence images of WT myofibers treated with agrin for 5 days immunostained for DHPR (red), a T-tubule marker, triadin (green), a junctional SR marker, and DAPI (blue). On the right are 2× magnifications of rectangles. Bar, 15 μm.Representative immunofluorescence images of WT myofibers treated with agrin for 10 days immunostained for DHPR (red), triadin (green), and DAPI (blue). On the right are 2× magnifications of rectangles. Bar, 15 μm.Quantification of transversal triads in WT myofibers treated or untreated with agrin and differentiated for 5 or 10 days. Error bars, s.e.m., *n* = 3. *P*-values from *t*-test (untreated versus treated condition).Representative electron microscopy image of WT primary myofibers, transfected with *GAPDH* siRNA and treated with agrin for 10 days. Bar 1 µm.Magnification of the rectangle in (F). Arrows indicate triads located between myofibrils at the level of A–I border. Bar 500 nm. Inset: high magnification of the right lower triad. Arrowheads indicate the RyR feet. Bar 100 nm. SR: sarcoplasmic reticulum.

During myofiber maturation, a complex network of signaling and cytoskeletal proteins operate to organize cellular structures such as contractile myofibrils and triads that allow EC coupling. In parallel, nuclei move from the center of the myofiber to the periphery (Franzini-Armstrong, 1986; Flucher *et al*, 1991; Starr & Fridolfsson, 2010; Metzger *et al*, 2012). Multiple myogenic cell lines along with primary myoblasts will readily differentiate into myotubes in culture (Blau *et al*, 1985; Davis *et al*, 1987). Thus far, it has proven difficult to generate mature myofibers *in vitro* with T-tubules transversally paired with SR in striated transversal triads and peripheral nuclei (Flucher *et al*, 1991; Cooper *et al*, 2004; Cusimano *et al*, 2009). We therefore developed an *in vitro* system in which we used primary myoblasts isolated from WT or histone 2B-GFP (H2B-GFP) P5 mouse pups to generate mature myofibers (Hadjantonakis & Papaioannou, 2004). The H2B-GFP allowed us to visualize nuclei positioning by time-lapse microscopy (Fig 1A). Primary myoblasts were differentiated into myotubes and treated with agrin. Myotubes were then covered with a matrigel layer, as described in the Materials and Methods section and Supplementary Fig S1A. We observed the maturation of these myotubes treated with agrin by dual color phase-contrast/epi-fluorescence multi-positioning time-lapse microscopy over a period of 10 days (Fig 1A, Supplementary Movie S1). Myotubes elongated and their nuclei migrated and rotated throughout the length of the myotube during differentiation. Nuclear movements decreased during differentiation, and nuclei became positioned at the periphery of the myofiber between day 5 and day 10 (Fig 1A). We also observed migration of mononucleated cells, sometimes touching the myofibers, although we never observed fusion between mononucleated cells and differentiating myofibers after agrin addition. After 10 days, we noted increased myofiber detachment and death.

To quantify the percentage of peripheral nuclei observed during myofiber maturation, cell cultures from WT and H2B-GFP mice were fixed 5 and 10 days after agrin addition and immunostained for dihydropyridine receptor (DHPR) and triadin. DHPR, a voltage-gated channel, is found at the T-tubules. Triadin, an adaptor protein, is found at the junctional SR compartment (Flucher *et al*, 1991; Marty *et al*, 2000). DHPR and triadin are not expressed in mononucleated myoblasts (Marty *et al*, 2000; Zheng *et al*, 2002) which allowed us to distinguish between peripheral nuclei in myofibers from nuclei in mononucleated cells attached to the myofibers (Supplementary Fig S1B). Five days after agrin addition, about 20% of nuclei were observed at the periphery in either WT or H2B-GFP myofibers (Fig 1B). DHPR and triadin were found throughout the cytoplasm in clusters (Fig 1C). Ten days after the addition of agrin, 70% of nuclei were positioned at the periphery of the myofiber (Fig 1B and D). Remarkably, we observed that DHPR and triadin were organized in a striated pattern of triads (Fig 1D). Hence, the percentage of fibers with organized triads was also measured 5 and 10 days after agrin addition. We scored 20% of myofibers with organized triads 5 days after agrin addition and up to 80% of myofibers with transversal triads 10 days after agrin addition (Fig 1E). Electron microscopy (EM) confirmed the organization of triads transversally with one T-tubule coupled with two terminal cisternae of the SR and ryanodine receptor (RyR) feet (Fig 1F and G). Finally, we analyzed myofibrillogenesis during myofiber maturation and found that myofibrils (visualized by co-staining with α-actinin and F-actin) were already formed 5 days after agrin addition (Supplementary Fig S1C). Therefore, our *in vitro* conditions were sufficient to generate mature myofibers with peripheral nuclei as well as T-tubules and SR organized in striated transversal triads.

### Amph2, dynamin 2, and myotubularin are involved in the peripheral positioning of nuclei and triad organization

**Figure 2:**
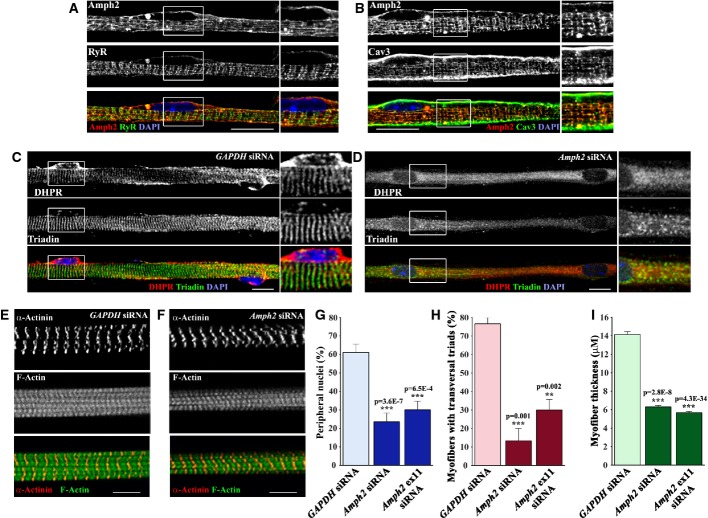
Amph2 is required for triad formation and peripheral localization of nuclei in *in vitro* myofibers Representative immunofluorescence images of WT primary myofibers treated with agrin for 10 days and immunostained for amph2 (red), RyR (green), and DAPI (blue). Bar, 15 μm.Representative immunofluorescence images of WT primary myofibers treated with agrin for 10 days and immunostained for amph2 (red), Cav3 (green), and DAPI (blue). Bar, 15 μm.Representative immunofluorescence images of WT primary myofibers transfected with *GAPDH* siRNA, treated with agrin for 10 days, and immunostained for DHPR (red), triadin (green), and DAPI (blue). On the right 2× magnifications of rectangles. Bar, 15 μm.Representative immunofluorescence images of WT primary myofibers transfected with *Amph2* siRNA, treated with agrin for 10 days, and immunostained for DHPR (red), triadin (green), and DAPI (blue). On the right 2× magnifications of rectangles. Bars, 15 μm.Representative immunofluorescence images of WT primary myofibers transfected with *GAPDH* siRNA, treated with agrin for 10 days, and immunostained for α-actinin (red), F-Actin (green). Bar, 15 μm.Representative immunofluorescence images of WT primary myofibers transfected with *Amph2* siRNA, treated with agrin for 10 days, and immunostained for α-actinin (red), F-Actin (green). Bar, 15 μm.Quantification of peripheral nuclei in myofibers transfected with *GAPDH*, *Amph2* siRNA, or *Amph2 ex11* siRNA, and treated with agrin for 10 days. Error bars, s.e.m., *n* = 3. *P*-values from *t*-test (*GAPDH* siRNA versus *Amph2* siRNA condition).Quantification of transversal triads in myofibers transfected with *GAPDH*, *Amph2* siRNA, or *Amph2 ex11* siRNA and treated with agrin for 10 days. Error bars, s.e.m., *n* = 3. *P*-values from *t*-test (*GAPDH* siRNA versus *Amph2* siRNA condition).Quantification of myofiber thickness in *GAPDH* siRNA, *Amph2* siRNA, or *Amph2 ex11* siRNA and treated with agrin for 10 days. Error bars, s.e.m., *n* = 3. *P*-values from *t*-test (*GAPDH* siRNA versus *Amph2* siRNA condition).

Amph2 is involved in the formation of T-tubules and is localized to transversal triads in adult myofibers (Butler *et al*, 1997; Lee *et al*, 2002). We investigated the localization of amph2 in *in vitro* myofibers 10 days after agrin addition. We observed that amph2 was organized in tubular structures both longitudinally and transversally throughout myofibers (Fig 2A and B). These structures co-localized with RyR1, found at the junctional SR (Zalk *et al*, 2007) and caveolin-3, found at the T-tubules and sarcolemma (Parton *et al*, 1997). Thus, amph2 also localizes to the organized striated triads in newly differentiated myofibers.

To determine a role for amph2 in nuclear positioning and transversal triad organization during myofiber differentiation, we transfected myoblasts with *Amph2* exon 3 siRNA, *Amph2* exon 11 siRNA, or *GAPDH* siRNA (as control) (Fig 2C and D, Supplementary Figs S2A and S4A). *Amph2* exon 3 is expressed in all amph2 isoforms, whereas *Amph2* exon 11 is specifically expressed in skeletal muscle isoforms (Nicot *et al*, 2007). Upon transfection, myoblasts were differentiated into myotubes and agrin was added 24 h after (Supplementary Fig S1A). Ten days after agrin addition, the number of nuclei per myofiber (fusion index) was similar in all experimental conditions (Supplementary Fig S2P). The levels of amph2 expression were strongly reduced in both *Amph2* exon 3 and exon 11 siRNA when compared to *GAPDH* siRNA (Supplementary Figs S2A–C and S4A). We found that nuclear positioning at the myofiber periphery, formation of transversal triads, and myofiber thickness were significantly inhibited in either *Amph2* exon3 or exon11 siRNAs when compared to non-transfected or *GAPDH* siRNA transfected myofibers (Fig 2C, D, G–I and Supplementary Fig S4A–C). Reducing the expression of dynamin 2 and myotubularin, encoded by genes mutated in CNM, also leads to a decrease in peripheral nuclei, myofibers with organized triads and myofiber thickness (Supplementary Fig S2D–O).

To study whether amph2 was involved in the regulation of myofibrillogenesis, we examined the morphology of myofibrils and found no differences between *Amph2* siRNA myofibers and *GAPDH* siRNA myofibers (Fig 2E and F). We also evaluated the microtubule cytoskeleton in *Amph2* siRNA myofibers, since microtubules are involved in nuclear positioning (Metzger *et al*, 2012). We did not observe any changes in the morphology of microtubules and in the expression of β-tubulin on *Amph2* siRNA myofibers when compared with *GAPDH* siRNA (Supplementary Fig S1D–G).

Alterations in triad structure and organization have been related to defects in intracellular calcium homeostasis after membrane depolarization (Powell *et al*, 1996). A role for amph2 in calcium homeostasis has been described in isolated muscle fibers after *Amph2* shRNA electroporation *in vivo* (Tjondrokoesoemo *et al*, 2011). To determine whether amph2 is also involved in calcium homeostasis during myofiber formation, we measured cytoplasmic calcium concentration in myofibers 10 days after agrin addition, using a fluorescent Fluo-4 direct calcium sensor (Taneike *et al*, 2011). We observed a reduction of cytoplasmic calcium influx after KCl-induced membrane depolarization or caffeine-induced calcium release from the sarcoplasmic reticulum in *Amph2* siRNA myofibers, when compared to *GAPDH* siRNA (Supplementary Fig S3A–C). These results suggest that amph2 is involved in calcium homeostasis during myofiber formation,

Finally, we tested the role of *AMPH2* mutations (associated with ARCNM) in nuclear positioning and triad organization. We transfected myotubes with cDNA encoding GFP-tagged amph2 mutated in the BAR domain (amph2 R154Q) and in the SH3 domain (amph2 K575X) (Toussaint *et al*, 2011). We found that expression of either R154Q or K575X amph2 mutants inhibited nuclear positioning at the periphery of myofibers and disrupted the organization of DHPR (Supplementary Fig S3D–G). We also observed a decrease in the thickness of the myofibers transfected with amph2 R154Q or amph2 K575X when compared to the GFP-transfected controls (Supplementary Fig S3H). Overall, these results reveal that amph2, dynamin 2, and myotubularin regulate nuclear peripheral positioning and triad organization during myofiber formation and that these functions are also disrupted by mutations in amph2 associated with ARCNM.

### N-WASP associates with amph2 in myofibers and is involved in nuclear localization and triad organization

**Figure 3:**
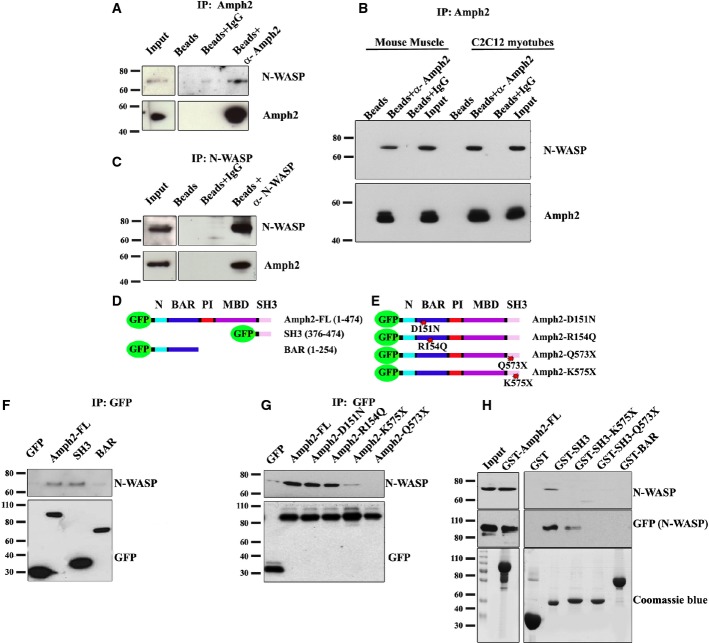
Amph2 interacts with N-WASP and the interaction is disrupted by CNM-associated amph2 mutations A Western blot with N-WASP and amph2 antibodies of endogenous amph2 immunoprecipitation in primary myofibers, treated with agrin for 10 days. B Western blot with N-WASP and amph2 antibodies of endogenous amph2 immunoprecipitation in mouse muscle or C2C12 myotubes. C Western blot with N-WASP and amph2 antibodies of endogenous N-WASP immunoprecipitation in primary myofibers. D Diagrams of the amph2 constructs used in (F). All constructs are N-terminally tagged with GFP. Numbers are amino acid position. E Diagrams of the amph2 full-length CNM mutants used in (G). Point mutation position is highlighted by a star. All constructs are N-terminal tagged with GFP. F, G Western blot with N-WASP and GFP antibodies of GFP immunoprecipitation in C2C12 cells expressing the indicated constructs. H GST pull down of N-WASP using the indicated GST-tagged amph2 proteins from muscle homogenates (top) or *in vitro* transcribed/translated GFP-N-WASP (middle). Coomassie blue staining of loaded gel is shown at the bottom. Source data are available online for this figure.

N-WASP interacts and regulates amphiphysin orthologs in *D. melanogaster* and *S. cerevisiae*, and amphiphysin-1 in mammalian brain (Madania *et al*, 1999; Salazar *et al*, 2003; Zelhof & Hardy, 2004; Yamada *et al*, 2009). Thus, we hypothesized that N-WASP could interact with amph2 in skeletal muscle and play a role in myofiber maturation and amph2-dependent ARCNM phenotype. We found that N-WASP co-immunoprecipitated with endogenous amph2 from extracts of mouse primary mature myofibers and C2C12 myotubes (Fig 3A and B). Conversely, amph2 co-immunoprecipitated with endogenous N-WASP from extracts of primary mature myofibers (Fig 3C). To identify the amph2 domain involved in the interaction with N-WASP, we expressed GFP-tagged full-length human amph2, SH3, or BAR domains of amph2 in C2C12 myoblasts (Fig 3D). We observed that GFP antibodies co-immunoprecipitated endogenous N-WASP together with amph2 SH3 domain or full-length amph2 (Fig 3F). We observed a very weak signal in BAR domain amph2 after co-immunoprecipitation, probably due to dimerization amph2 via the BAR domain (Lee *et al*, 2002; Friesen *et al*, 2006). In addition, we purified human GST-tagged full-length SH3 or BAR domains of amph2 and found that the SH3 domain and the full-length amph2 pulled down N-WASP from muscle homogenates or from *in vitro* transcribed/translated GFP-N-WASP (Fig 3H). These results demonstrate that the SH3 domain of amph2 associates with N-WASP in skeletal muscle and that likely this interaction is direct.

We next tested whether the *AMPH2* mutations found in ARCNM patients could disrupt the interaction of amph2 with N-WASP. We expressed human GFP-tagged amph2 with BAR domain mutations (D151N and R154Q) or SH3 domain mutations (Q573X and K575X) in C2C12 cells (Fig 3E). We observed that amph2 with BAR domain mutations co-immunoprecipitated with the same amount of N-WASP as full-length amph2 (Fig 3G). In contrast, amph2 SH3 mutation Q573X and amph2-K575X did not co-immunoprecipitate with endogenous N-WASP. In addition, we expressed and purified GST-tagged amph2 SH3 domain with the same Q573X and K575X mutations and found that these proteins did not pull down N-WASP from muscle homogenates or from *in vitro* transcribed/translated GFP-N-WASP, except for SH3-K575X that pulled down a residual amount of *in vitro* transcribed/translated GFP-N-WASP (Fig 3H).

**Figure 4:**
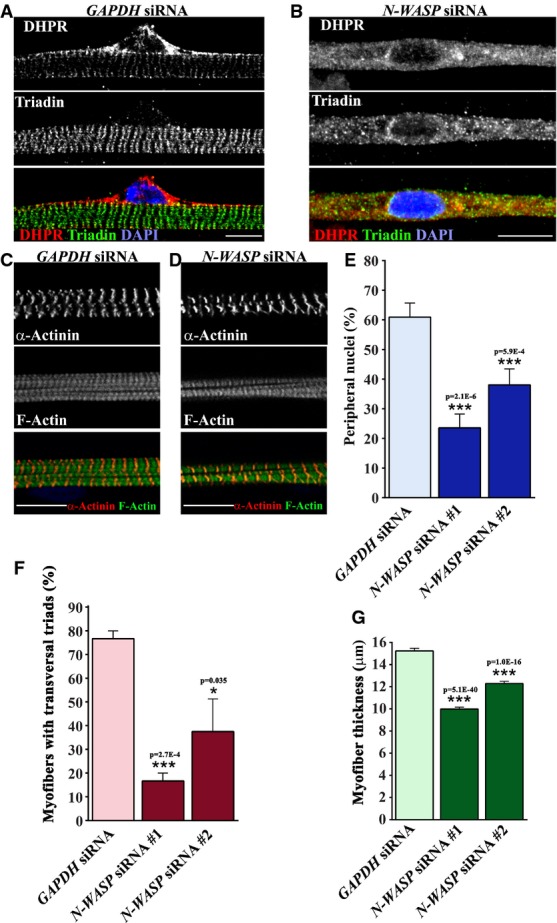
N-WASP is required for peripheral localization of nuclei and triad organization during myofiber formation Representative immunofluorescence images of WT primary myofibers transfected with *GAPDH* siRNA, treated with agrin for 10 days, and immunostained for DHPR (red), triadin (green), and DAPI (blue). Bar, 15 μm.Representative immunofluorescence images of WT primary myofibers transfected with *N-WASP* siRNA, treated with agrin for 10 days, and immunostained for DHPR (red), triadin (green), and DAPI (blue). Bars, 15 μm.Representative immunofluorescence images of WT primary myofibers transfected with *GAPDH* siRNA, treated with agrin for 10 days, and immunostained for α-actinin (red) and F-Actin (green). Bar, 15 μm.Representative immunofluorescence images of WT primary myofibers transfected with *N-WASP* siRNA, treated with agrin for 10 days, and immunostained for α-actinin (red) and F-Actin (green). Bar, 15 μm.Quantification of peripheral nuclei in myofibers transfected with *GAPDH* or *N-WASP* siRNA and treated with agrin for 10 days. Error bars, s.e.m., *n* = 3. *P*-values from *t*-test.Quantification of transversal triads in myofibers transfected with *GAPDH* or *N-WASP* siRNA and treated with agrin for 10 days. Error bars, s.e.m., *n* = 3. *P*-values from *t*-test.Quantification of myofiber thickness in myofibers transfected with *GAPDH* or *N-WASP* siRNA and treated with agrin for 10 days. Error bars, s.e.m., *n* = 3. *P*-values from *t*-test.

Finally, we tested the role of N-WASP on peripheral positioning of nuclei and triad organization during myofiber differentiation. We efficiently reduced the levels of N-WASP in myotubes using siRNA without observing any changes in fusion index when compared with *GAPDH* siRNA (Supplementary Figs S2P and S3I and J). We observed a reduction of peripheral nuclear positioning, triad organization, and myofiber thickness in *N-WASP* siRNA myofibers (Fig 4A, B, E–G). Myofibrils morphology was not affected when compared to *GAPDH* siRNA myofibers (Fig 4C and D). Altogether, these results suggest a role for N-WASP in peripheral nuclei positioning and triad organization, probably mediated by N-WASP interaction with amph2. Moreover, the disruption of amph2 and N-WASP interaction by SH3 domain mutations found in ARCNM suggests that alteration of N-WASP function participates in the etiology of the disease.

### N-WASP functions downstream of amph2 to regulate nuclear localization and triad organization

**Figure 5:**
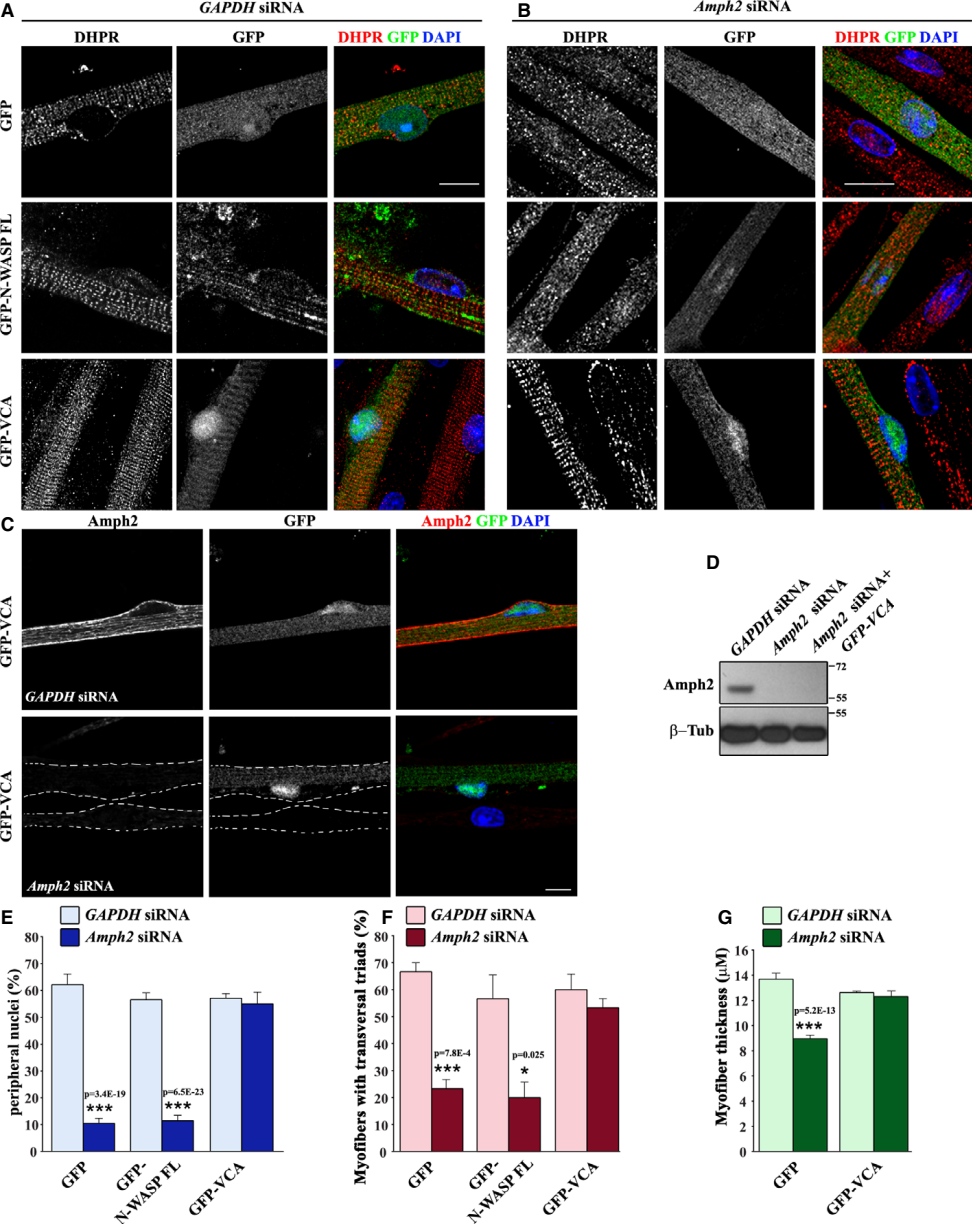
N-WASP involvement downstream of amph2 Representative immunofluorescence images of myofibers transfected with *GAPDH* siRNA and GFP (top), GFP-N-WASP full-length (N-WASP FL, middle) or GFP-VCA (VCA, bottom), treated with agrin for 10 days, and stained for DHPR (red) and DAPI (blue). Bar, 15 μm.Representative immunofluorescence images of myofibers transfected with *Amph2* siRNA and GFP (top), GFP-N-WASP (middle), or GFP-VCA (bottom), treated with agrin for 10 days and stained for DHPR (red) and DAPI (blue). Bar, 15 μm.Representative immunofluorescence images of myofibers transfected with GFP-VCA and *GAPDH* siRNA (top) or *Amph2* siRNA (bottom), and stained for amph2 (red) and DAPI (blue). Bar, 15 μm.Western blot with amph2 and β-tubulin antibodies of myofibers transfected with *Amph2* siRNA or *Amph2* siRNA and GFP-VCA.Quantification of peripheral nuclei in myofibers transfected with *GAPDH* (light blue) or *Amph2* (blue) siRNA and with GFP, GFP-N-WASP-FL, or GFP-VCA, and treated with agrin for 10 days. Error bars, s.e.m., *n* = 3. *P*-values from *t*-test (GFP versus GFP-N-WASP-FL or GFP-VCA).Quantification of transversal triads in myofibers transfected with *GAPDH* (pink) or *Amph2* (red) siRNA and with GFP, GFP-N-WASP-FL, or GFP-VCA, and treated with agrin for 10 days. Error bars, s.e.m., *n* = 3. *P*-values from *t*-test (GFP versus GFP-N-WASP-FL or GFP-VCA).Quantification of myofiber thickness in myofibers transfected with *GAPDH* (light green) or *Amph2* (green) siRNA and with GFP and GFP-VCA, and treated with agrin for 10 days. Error bars, s.e.m., *P*-values from *t*-test (GFP versus GFP-VCA conditions). Source data are available online for this figure.

Our results demonstrating a physical interaction between amph2 and N-WASP in myofibers, and the similar phenotypes observed when N-WASP or amph2 are depleted in myofibers, suggest that N-WASP functions together with amph2 to regulate peripheral nuclear localization and triad organization. N-WASP activity is modulated by the switch from an auto-inhibited configuration to an activated state by different mechanisms; among them is the binding of SH3 domains to the polypro region of N-WASP. Once activated, the C-terminal VCA domain of N-WASP interacts with the Arp2/3 complex, leading to actin polymerization (Prehoda *et al*, 2000; Padrick & Rosen, 2010). To determine whether N-WASP functions downstream of amph2, we expressed full-length N-WASP or the constitutively active VCA domain of N-WASP in *Amph2* or *GAPDH* siRNA myotubes. After 10 days of differentiation in the presence of agrin, we found that expression of the VCA domain of N-WASP in *Amph2* exon 3 or exon 11 siRNA myofibers restored peripheral localization of nuclei, triad organization as well as myofiber thickness to the same level as the *GAPDH* siRNA (Fig 5A, B, E–G and Supplementary Fig S4A–C). Expression of full-length N-WASP or GFP in *Amph2* siRNA myofibers was not able to restore any of these phenotypes, suggesting that amph2 is required to activate N-WASP. Furthermore, the expression of either GFP, full-length N-WASP, or VCA domain in *GAPDH* siRNA myofibers did not interfere with peripheral localization of nuclei, triad organization, or myofiber thickness (Fig 5A, E–G, and Supplementary Fig S4B and C). To rule out that the rescue effect we observed upon GFP-VCA expression in *Amph2* siRNA myofibers was not due to an indirect role of N-WASP on gene expression (Wu *et al*, 2006), we tagged the VCA domain with TdTomato (TdT-VCA), a 60-kD fluorescent protein, to prevent the accumulation of the VCA domain in the nucleus (Supplementary Fig S4D). We found that expression of TdT-VCA in *Amph2* siRNA myofibers also restored peripheral localization of nuclei and triad organization (Supplementary Fig S4E and F). Furthermore, we observed that amph2 was not detectable by Western blot or immunofluorescence analysis in myofibers transfected with GFP-VCA and *Amph2* exon 3 or exon 11 siRNA (Fig 5C and D, and Supplementary Fig S4A). Therefore, expression of VCA does not induce expression of amph2 in *Amph2* siRNA myofibers. Finally, we also tested whether N-WASP could function downstream of myotubularin and dynamin 2. We expressed GFP-VCA in myofibers depleted for myotubularin or dynamin 2. We found that GFP-VCA expression rescued peripheral nuclear positioning and triad organization in *dynamin 2* siRNA myofibers, but not *myotubularin* siRNA myofibers (Supplementary Fig S2M and N).

Overall, these results indicate that N-WASP functions downstream of amph2 and dynamin 2 to regulate peripheral nuclear localization and triad organization formation during myofiber maturation.

### Nuclear localization and triad organization are regulated by independent pathways

**Figure 6:**
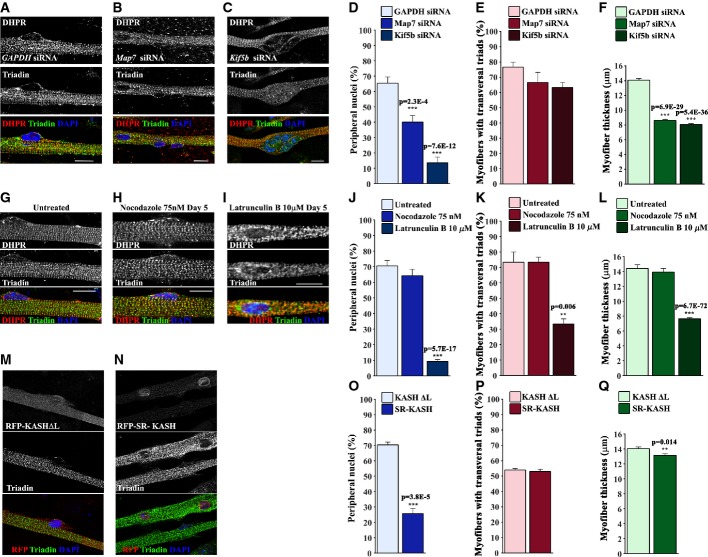
Role of microtubules and actin in nuclear positioning and triad organization during myofiber maturation A–C Representative immunofluorescence images of WT primary myofibers transfected with *GAPDH* siRNA (A), *Map7* siRNA (B), or K*if5b* siRNA (C), treated with agrin for 10 days and subsequently immunostained for DHPR (red), triadin (green), and DAPI (blue). Bar, 15 μm. D Quantification of peripheral nuclei in myofibers transfected with *GAPDH, Map7,* or *Kif5b* siRNA. Error bars, s.e.m., *n* = 3. *P*-values from *t*-test. E Quantification of transversal triads in myofibers transfected with *GAPDH, Map7,* or *Kif5b* siRNA. Error bars, s.e.m., *n* = 3. *P*-values from *t*-test. F Quantification of myofiber thickness in myofibers transfected with *GAPDH, Map7,* or *Kif5b* siRNA. Error bars, s.e.m., *n* = 3. *P*-values from *t*-test. G–I Representative immunofluorescence images of WT primary myofibers untreated (G) or treated with 75 nM of nocodazole (H) or 10 μM of latrunculin B (I) at day 5 after agrin treatment, and immunostained at day 10 for DHPR (red), triadin (green), and DAPI (blue). Bar, 15 μm. J Quantification of peripheral nuclei in myofibers untreated or treated with nocodazole and latrunculin B as described in (G–I). Error bars, s.e.m., *n* = 3. *P*-values from *t*-test. K Quantification of transversal triads in myofibers untreated or treated with nocodazole and latrunculin B as described in (G–I). Error bars, s.e.m., *n* = 3. *P*-values from *t*-test. L Quantification of myofiber thickness in myofibers untreated or treated with nocodazole and latrunculin B as described in (G–I). Error bars, s.e.m., *n* = 3. *P*-values from *t*-test. M, N Representative immunofluorescence images of WT primary myofibers transfected with RFP-KASHΔL (M) and RFP-SR-KASH (N), 10 days after agrin addition immunostained for triadin (green) and DAPI (blue). Bar, 15 μm. O Quantification of peripheral nuclei in myofibers transfected with RFP-KASHΔL or RFP-SR-KASH as described in (M, N). Error bars, s.e.m., *n* = 3. *P*-values from *t*-test. P Quantification of transversal triads in myofibers transfected with RFP-KASHΔL or RFP-SR-KASH as described in (M, N). Error bars, s.e.m., *n* = 3. *P*-values from *t*-test. Q Quantification of myofibers thickness in myofibers transfected with RFP-KASHΔL or RFP-SR-KASH as described in (M, N). Error bars, s.e.m., *n* = 3. *P*-values from *t*-test.

Our results suggest that nuclear positioning at the periphery of the myofiber is dependent on N-WASP. We next decided to investigate how N-WASP controls peripheral nuclear positioning. We previously showed that the spreading of nuclei along the myofibers is dependent on a conserved mechanism involving Map7, a microtubule-binding protein, Kif5b, a kinesin, and microtubules (Metzger *et al*, 2012). Thus, since spreading of nuclei along the myofibers precedes peripheral nuclear positioning (Fig 1A), we therefore explored the involvement of Map7/Kif5b/microtubule pathway on nuclear positioning at the periphery of myofibers and triad organization. We measured peripheral nuclear position and triad organization in myofibers depleted for Map7 and Kif5b using siRNA. We observed that nuclei in *Map7* and *Kif5b* siRNA myofibers were not spread along the myofibers as we previously described (Fig 6B and C) (Metzger *et al*, 2012; Wang *et al*, 2013). We also found that peripheral nuclear positioning and myofiber thickness were reduced in *Map7* and *Kif5b* siRNA myofibers. Remarkably, triad organization was not impaired (Fig 6A–F).

At day 5 after agrin addition, nuclei in the myofibers are already spread along the myofiber, but are not yet positioned at the periphery of the myofiber (Fig 1A–C, Supplementary Movie S1). Thus, the inhibition of peripheral nuclear positioning could be due to an impairment of the spreading of nuclei along the myofiber or due to the direct involvement of the Map7/Kif5b/microtubule pathway on peripheral nuclear positioning. To distinguish between these two hypothesis, we treated myofibers at day 5 after agrin addition with low dose of nocodazole (75 nM), which alters microtubule dynamics required for microtubule-dependent nuclear movement (Cadot *et al*, 2012) and allows us to test whether microtubules are directly involved in peripheral nuclear positioning. We found that inhibition of microtubule dynamics after day 5 of agrin addition did not prevent peripheral nuclear positioning (Fig 6H and J). We also did not observe any alterations in triad organization or myofiber thickness (Fig 6K and L). Overall, these results suggest that peripheral nuclear positioning is not dependent on microtubules but requires Map7/Kif5b/microtubule-dependent spreading of nuclei along the myofiber prior to nuclear positioning at the periphery of the fiber. Furthermore, triad organization is not dependent on nuclear positioning.

Our results suggest that nuclear positioning at the periphery of the myofiber is dependent on N-WASP, an actin regulator (Fig 4). Nesprins are a class of KASH-domain nuclear envelope proteins that bind to actin and are involved in the position of the nucleus in matured myofibers (Grady *et al*, 2005). We previously showed that the spreading of nuclei along myofibers is independent of nesprins, therefore nesprins are probably involved in nuclear positioning events that occur after nuclei spreading in the myofibers (Metzger *et al*, 2012). To test this hypothesis, we expressed in myoblasts a dominant-negative KASH construct (SR-KASH) that removes KASH proteins from the nuclear envelope and disrupts the connection between the nucleus and the actin cytoskeleton (Grady *et al*, 2005). We also expressed a control KASH construct lacking the C-terminal region required for nuclear envelope anchoring (KASHΔL) (Luxton *et al*, 2010). We observed that expression of SR-KASH inhibited peripheral nuclear position 10 days after agrin addition, without any effect on triad organization when compared with control KASHΔL (Fig 6M–P). In addition, expression of SR-KASH induced a minor, although significant, decrease of myofiber thickness, when compared with KASHΔL (Fig 6Q). These results suggest that nesprins are involved in peripheral nuclear positioning but not in triad organization. Finally, to determine whether actin is also involved in peripheral nuclear positioning, we treated myofibers at day 5 after agrin addition with latrunculin B to impair actin dynamics. We observed that peripheral nuclear positioning, triad organization, and fiber thickness were reduced 10 days after agrin addition (Fig 6I–L), suggesting that actin is involved in peripheral nuclear positioning and triad organization. Overall, our results demonstrate that peripheral nuclear positioning is dependent on the correct spreading of nuclei along the myofiber driven by Map7/Kif5b/microtubules pathway, on nesprins and on actin. Furthermore, we show that triad organization is not dependent on the position of the nucleus at the periphery of the myofiber.

### N-WASP and amph2 are involved in the maintenance of triad organization in skeletal muscle

**Figure 7:**
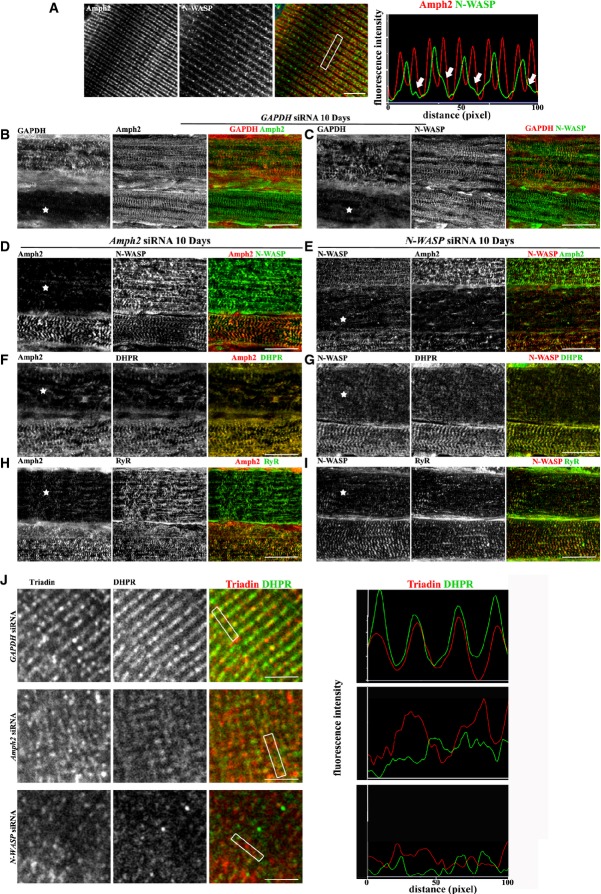
N-WASP and amph2 localization in adult muscle and its role on the maintenance of triad organization Left panel: representative immunofluorescence images of isolated WT mouse muscle fibers immunostained for amph2 (red) or N-WASP (green). Right panel: line-scan of indicated region in the left panels showing average intensity of N-WASP (green) and amph2 (red), respectively. Bar, 1 μm.Representative immunofluorescence images of longitudinal sections of WT mouse muscle electroporated with *GAPDH* siRNA and immunostained after 10 days for GAPDH (red) and amph2 (green). A star indicates a myofiber depleted for GAPDH. Bar, 50 μm.Representative immunofluorescence images of longitudinal sections of WT mouse muscle electroporated with *GAPDH* siRNA and immunostained after 10 days for GAPDH (red) and N-WASP (green). A star indicates a myofiber depleted for GAPDH. Bar, 50 μm.Representative immunofluorescence images of longitudinal sections of WT mouse muscle electroporated with *Amph2* siRNA and immunostained after 10 days for amph2 (red), N-WASP (green). A star indicates a myofiber depleted for amph2. Bar, 50 μm.Representative immunofluorescence images of longitudinal sections of WT mouse muscle electroporated with *N-WASP* siRNA and immunostained after 10 days for N-WASP (red), and amph2 (green). A star indicates a myofiber depleted for N-WASP. Bar, 50 μm.Representative immunofluorescence images of longitudinal sections of WT mouse muscle electroporated with *Amph2* siRNA and immunostained after 10 days for amph2 (red), DHPR (green). A star indicates a myofiber depleted for amph2. Bar, 50 μm.Representative immunofluorescence images of longitudinal sections of WT mouse muscle electroporated with *N-WASP* siRNA and immunostained after 10 days for, N-WASP (red) and DHPR (green). A star indicates a myofiber depleted for N-WASP. Bar, 50 μm.Representative immunofluorescence images of longitudinal sections of WT mouse muscle electroporated with *Amph2* siRNA and immunostained after 10 days for amph2 (red) and RyR (green). A star indicates a myofiber depleted for amph2. Bar, 50 μm.Representative immunofluorescence images of longitudinal sections of WT mouse muscle electroporated with *N-WASP* siRNA and immunostained after 10 days for N-WASP (red), and RyR (green). A star indicates a myofiber depleted for N-WASP. Bar, 50 μm.Left panels: representative immunofluorescence images of isolated mouse muscle fibers, immunostained for triadin (red) and DHPR (green) in *GAPDH* siRNA, *Amph2* siRNA, and *N-WASP* siRNA electroporated fibers, respectively. Right panel: line-scan of indicated regions in the left panel showing average intensity of N-WASP staining (green) compared to amph2 (red), respectively. Bar, 1 μm.

**Figure 8:**
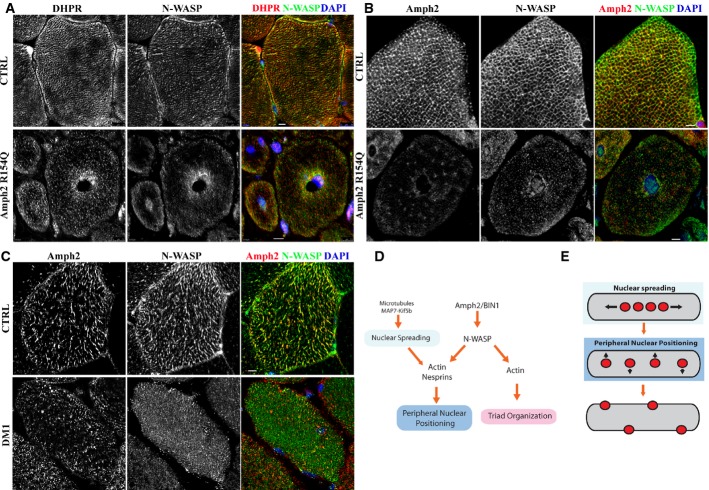
N-WASP and amph2 localization in muscle from healthy and ARCNM patients Representative immunofluorescence images of transversal sections of human muscle from healthy donor (top) or ARCNM patient carrying AMPH2 R154Q mutation, immunostained for DHPR (red), N-WASP (green), and DAPI (blue). Bars, 15 μm.Representative immunofluorescence images of transversal sections of human muscle from healthy donor (top) or ARCNM patient carrying AMPH2 R154Q mutation, immunostained for amph2 (red), N-WASP (green), and DAPI (blue). Bars, 15 μm.Representative immunofluorescence images of transversal sections of human muscle from healthy donor (top) or DM1 patient, immunostained for amph2 (red), N-WASP (green), and DAPI (blue). Bars, 15 μm.Schematic representation of the pathways that regulate triad organization and peripheral nuclear position downstream of amph2 and N-Wasp.Model for peripheral nuclear positioning during myofiber formation. After fusion of myoblast, nuclei (red) of myotubes cluster in the center of the myotube before nuclear spreading. After nuclei spread along the myotube, they become peripheral located at the myofiber periphery.

Amph2 is localized specifically at striated triads, whereas N-WASP is mainly found at the Z-line and also in discrete patches between Z-lines in longitudinal sections of mature muscle fibers (Butler *et al*, 1997; Razzaq *et al*, 2001; Takano *et al*, 2010) (Supplementary Fig S5B). Since striated triads are found primarily between Z-lines (Flucher, 1992) (Supplementary Fig S5D), we hypothesized that the discrete patches of N-WASP observed between Z-lines could localize to the triads. We determined the localization of N-WASP in adult mouse isolated fibers and observed that the discrete patches of N-WASP co-localized with amph2 and DHPR (Fig 7A and Supplementary Fig S5C). In transversal sections of adult mouse and human muscle fibers, we observed strong co-localization between N-WASP and DHPR (Fig 8 and Supplementary Fig S5A), suggesting that N-WASP is also found at the triads in mature muscle fibers. Finally, we analyzed human muscle biopsies by differential centrifugation (Gokhin & Fowler, 2011) and found that N-WASP was present in the same cellular membrane fractions as amph2 and DHPR (Supplementary Fig S8A and B). To determine when N-WASP is recruited to the triads, we analyzed the localization of N-WASP during myofiber formation *in vitro*. We found that 10 days after agrin addition, N-WASP starts to accumulate at the newly formed striated triads (Supplementary Fig S5E). Overall these results strongly suggest that a pool of N-WASP is also localized to the triads in newly formed and matured myofibers.

We next determined whether the localization of N-WASP in adult muscle fibers was dependent on amph2. We electroporated *Amph2* or *GAPDH* siRNA, together with a plasmid encoding the fluorescent protein TdT, in adult mouse muscle. We collected whole muscle or isolated single muscle fibers expressing TdT 10 days after electroporation (Supplementary Fig S6A) and immunostained for amph2 and N-WASP either in muscle sections or in isolated fibers. The distribution of amph2 and N-WASP was not affected in *GAPDH* siRNA muscle sections (Fig 7B and C) or isolated fibers (Supplementary Fig S6B) compared to non-transfected controls. Importantly, N-WASP distribution was disrupted in *Amph2* depleted myofibers (Fig 7D and Supplementary Fig S7B) and myofibril morphology was not altered, as previously reported (Supplementary Fig S7A) (Toussaint *et al*, 2011).

We next determined the involvement of N-WASP in the distribution of amph2. We collected whole muscle and isolated fibers from muscles electroporated with *N-WASP* siRNA and observed that amph2 distribution was disrupted (Fig 7E and Supplementary Fig S6B). No changes in myofibril morphology were detected in *N-WASP* siRNA myofibers, when compared to *GAPDH* siRNA myofibers, as previously reported (Supplementary Fig S7A) (Takano *et al*, 2010). Interestingly, nuclei remained at the periphery of the myofibers depleted for amph2 and N-WASP, suggesting that these proteins are not involved in anchoring the nuclei at the periphery of muscle fibers in adult skeletal muscle at least up to 10 days after electroporation (data not shown). Finally, we tested the role of *AMPH2* ARCNM mutations on the distribution of N-WASP in adult muscle. We electroporated GFP-tagged amph2 with a BAR domain (D151N) or a SH3 domain (K575X) mutations into adult muscle and isolated single muscle fibers positive for GFP expression (Supplementary Fig S6C). We found that both mutations disrupted the localization of N-WASP (Supplementary Fig S6D). Overall, these results suggest that amph2 is involved in the distribution of N-WASP in muscle. Conversely, N-WASP is also involved in the distribution of amph2 in muscle.

Our results show that amph2 and N-WASP are involved in the organization of triads during myofiber maturation (Figs2 and 4). Thus, we next determined the involvement of amph2 and N-WASP in the maintenance of organized triads in adult muscle. We electroporated *Amph2, N-WASP,* or *GAPDH* siRNA into adult mouse muscle and analyzed the distribution of DHPR and RyR1 in longitudinal muscle sections. Knockdown of *Amph2* or *N-WASP* in muscle fibers leads to disruption of DHPR distribution (Fig 7F and G). RyR1 distribution was also disrupted in *N-WASP* siRNA fibers but less disrupted in *Amph2* siRNA fibers (Fig 7H and I). We also analyzed isolated single muscle fibers from muscle transfected with *Amph2* or *N-WASP* siRNA and observed a strong alteration of triad organization (Fig 7J). These results suggest a role for amph2 and N-WASP on the maintenance organized triads in adult muscle fibers.

### N-WASP distribution and expression is altered in centronuclear myopathy and myotonic dystrophy

Since our results support a role for N-WASP in amph2-associated ARCNM, we analyzed the localization of N-WASP in a muscle biopsy from an ARCNM patient carrying *AMPH2* mutation (R154Q), and compared it to a healthy muscle biopsy. In transversal sections of healthy muscle biopsy, N-WASP was found in a reticulated structure that co-localized with DHPR and amph2 (Fig 8A and B). However, N-WASP organization in ARCNM muscle biopsy was severely disrupted, with a significant accumulation around the centrally located nuclei (Fig 8A and B). DHPR and amph2 distribution was also severely impaired, as previously described (Fig 8A and B) (Toussaint *et al*, 2011). Analysis of the same muscle biopsies by differential centrifugation confirmed the changes in the distribution of N-WASP, amph2, DHPR, and Cav3, but not α-actinin (Supplementary Fig S8A and B). We also determined the localization of N-WASP, amph2, and DHPR in muscle biopsies from ADCNM and XLCNM patients carrying *DNM2* mutation (E368K) and *MTM1* mutation (R421InsFIG), respectively (Supplementary Fig S8C–F). In ADCNM patients, N-WASP, amph2, and DHPR exhibited a more diffused distribution, with accumulation around the centrally located nuclei similar to what was observed for ARCNM patients. On the other hand, the changes in distribution of N-WASP, amph2, and DHPR between XLCNM and healthy patient biopsies were milder.

The changes in organization of N-WASP could be caused by alterations of N-WASP expression in muscle. We measured mRNA levels of *N-WASP*, *DHPR*, *AMPH2*, *DNM2,* and *MTM1* in different CNM muscle biopsies and did not find any changes when compared with healthy biopsies (Supplementary Fig S9A), except for *MTM1* in XLCNM, as previously described (Vasli *et al*, 2012). Next, we measured protein levels in the same CNM muscle biopsies and found that N-WASP protein levels were decreased in ARCNM and ADCNM, but not in XLCNM (Supplementary Fig S9B and C). Therefore, mutations in *AMPH2* and *DNM2*, but not *MTM1*, can result in a reduction of the stability of N-WASP protein.

Amph2 is mis-spliced in myotonic dystrophy of type 1 (DM1), a muscular dystrophy with histopathological features such as centrally located nuclei without signs of regeneration, muscle fiber atrophy and T-tubules alterations, also observed in CNM (Fugier *et al*, 2011). Therefore, we analyzed the distribution of N-WASP and amph2 in transversal section of biopsies from patients with DM1. We found that N-WASP and amph2 distribution was disrupted, when compared with a healthy biopsy (Fig 8C). We also measured the levels of mRNA and did not detect a decrease of *N-WASP, AMPH2, DHPR, DNM2,* and *MTM1* mRNA in DM1 patient muscle samples (Supplementary Fig S9A). These results suggest that N-WASP might also be implicated in DM1 pathology.

## Discussion

In this work, we identified a role for N-WASP downstream of amph2 regulating the organization of triads and the position of the nucleus at the periphery of myofibers. Furthermore, we showed that nuclear positioning at the periphery of myofibers is dependent on nesprins, actin and requires prior distribution of nuclei along the myofibers, driven by microtubules, Map7 and Kif5b. In addition, triad organization occurs independently of nuclear positioning at the periphery of myofibers (Fig 8D and E). Finally, we provide evidence for the disruption of N-WASP function in centronuclear myopathies and myotonic dystrophies, probably downstream of amph2, suggesting an impairment of the identified molecular mechanism on these muscle disorders.

### An *in vitro* system suitable to study molecular mechanisms of myofiber maturation and muscle disorders

Previous reports described the formation of differentiated myofibers *in vitro*. Flucher *et al* (1994) described the organization of T-tubules in longitudinal orientation in mouse myotubes, although transversal organization was occasionally observed and peripheral localization of nuclei was not assessed. Cusimano *et al* (2009) observed the formation of aligned striated triads, but the peripheral localization of nuclei was not reported. Cooper *et al* (2004) reported peripheral positioning of nuclei in mouse myofibers, although triad organization was not studied. In this manuscript, we report a novel *in vitro* system to differentiate mouse myoblasts into mature myofibers with clearly observable striated transversal triads, myofibrils, and peripheral nuclei (Fig 1). The ultrastructure of triads and myofibrils of the *in vitro* myofibers was more developed than myofibers from neonatal mouse muscle since most of the triads were already transversally organized and we rarely observed diads (Ito *et al*, 2001; Takekura *et al*, 2001).

We used this system to identify the role of amph2 and N-WASP on triad organization and peripheral nuclear positioning, to identify the mechanism that drives peripheral nuclear positioning, and to describe that triad organization occurs independently of peripheral nuclear positioning. We also showed that mutations in amph2 associated with ARCNM disrupt the function of amph2 and N-WASP on nuclear positioning and triad organization. This system can be used to study molecular mechanisms that occur during myofiber formation from initial fusion events to later stages of the establishment of functional transversal triads. Furthermore, this system provides a platform to screen for potential drugs that will prevent myofiber defects associated with CNM and other muscle disorders.

### Involvement of amph2 and N-WASP in triad organization

The mechanisms of triad organization and maintenance are poorly understood. Formation and maintenance of membrane deformation of the T-tubules could be mediated by the actin cytoskeleton, which provides the required scaffold to stabilize membrane shape (Suetsugu & Gautreau, 2012). Amph2, a BAR domain-containing protein, is involved in T-tubule biogenesis and accumulates to T-tubules (Butler *et al*, 1997; Lee *et al*, 2002; Toussaint *et al*, 2011). Our data suggest that the SH3 domain of amph2 forms a complex with N-WASP that is important for triad organization (Figs3 and 4). Amph2 is probably involved in the localization of N-WASP to triads since amph2 BAR domain mutations perturb the distribution of N-WASP without disrupting the interaction between amph2 and N-WASP (Figs3G, 8 and Supplementary Fig S6D). In addition, the distribution of N-WASP in DM1 patient samples is also disturbed probably due to mislocalization of alternatively spliced amph2 (Fig 8C) (Fugier *et al*, 2011). We also provide evidence for a role of N-WASP downstream of amph2 regulating triad organization since we found that expression of constitutively active N-WASP restored triad organization in amph2-depleted myofibers (Fig 5 and Supplementary Fig S4).

We propose a model in which amph2 recruits N-WASP to the vicinity of T-tubules allowing N-WASP to regulate the actin cytoskeleton required for structural stability and organization of triads. The actin cytoskeleton that regulates the organization and maintenance of triads is unknown; however, gamma-actin is a good candidate. Gamma-actin accumulates in the vicinity of the triads (Kee *et al*, 2004, 2009) and co-fractions with SR proteins after differential centrifugation (Gokhin & Fowler, 2011). In addition, skeletal muscles of gamma-actin knockout mice progressively accumulate centrally located nuclei without any signs of regeneration (damaged sarcolemma, inflammation, and fibrosis), which is similar to what can be observed in CNM (Biancalana *et al*, 2003; Sonnemann *et al*, 2006; Al-Qusairi *et al*, 2009). In our model, N-WASP might regulate this pool of gamma-actin via the Arp2/3 complex or/and nebulin (Prehoda *et al*, 2000; Takano *et al*, 2010). Furthermore, tropomyosin Tm5NM1 was described to accumulate specifically at the triads, and Tm5NM1 knockout mice show defects in triad organization and EC coupling (Vlahovich *et al*, 2009). Tropomyosins are involved in actin filaments stabilization; therefore, we speculate that an equilibrium between actin dynamics regulated by N-WASP and actin stabilization regulated by tropomyosin might be involved in triad organization and maintenance, similar to what is observed in lamellipodium-based cell motility (Bugyi *et al*, 2010). N-WASP was recently found to regulate the polymerization of alpha-actin at the Z-lines of myofibrils (Takano *et al*, 2010). N-WASP is not required for the formation of myofibrils, however, is involved in myofibril maturation and myofiber growth (Takano *et al*, 2010) (Fig 4C, D and G). Therefore, we speculate that N-WASP could regulate two independent pools of actin (alpha and gamma) in two different myofiber structures (Z-lines and triads). A similar mechanism is probably present in cardiac muscle where an amph2-specific isoform is involved in T-tubule morphology (Hong *et al*, 2014). Future experiments in cardiac muscle should provide evidence for an interaction between amph2 and N-WASP and a role for N-WASP on T-tubule morphology.

### Molecular mechanism of nuclear positioning during myofiber formation

Nuclei are positioned at the periphery during myofiber differentiation, a process which is highly regulated (Bruusgaard *et al*, 2003; Starr & Fridolfsson, 2010). We can deduce that peripheral nuclear positioning during myofiber maturation is not simply the consequence of unspecific peripheral pushing forces due to sarcomere formation because: (1) sarcomeres are formed prior to peripheral nuclear positioning and (2) we do not observe any defects on sarcomere morphology when peripheral nuclear positioning is inhibited (Figs2, 4 and 6). Several mechanisms dependent on actin-binding proteins of the nuclear envelope (nesprins) and microtubules influence nuclear positioning during skeletal muscle differentiation (Zhang *et al*, 2007; Metzger *et al*, 2012). In this work, we establish that peripheral nuclear position requires two separate steps. First, nuclei must spread along the myofiber by a microtubule/Map7/Kif5b-dependent mechanism (Metzger *et al*, 2012). Second, nuclear movement to the periphery is dependent on amph2, N-WASP, actin, and nesprins (Fig 8D and E). Therefore, a switch from a microtubule-driven to an actin-driven nuclear positioning mechanism probably occurs during skeletal muscle differentiation. Finally, our results suggest that amph2, N-WASP, and actin are involved in both formation of triads and peripheral nuclear positioning. However, microtubules, Map7, Kif5, and nesprins are mainly involved in peripheral nuclear positioning with no identifiable role on the formation of triads.

### Amph2 and N-WASP involvement in muscle diseases

Centronuclear myopathies are characterized by centrally located nuclei and defects in T-tubules, triads, and excitation–contraction coupling (Al-Qusairi *et al*, 2009; Dowling *et al*, 2009; Toussaint *et al*, 2011). Our results strongly suggest that mutations in *AMPH2* and *DNM2*, but not *MTM1*, prevent N-WASP from accumulating at triads (Fig 8). A common molecular mechanism may thus link amph2 and dynamin 2 with N-WASP, as previously proposed (Cowling *et al*, 2012). N-WASP function in CNM is probably impaired by disruption of N-WASP targeting to the membranes of T-tubules due to mutations in *AMPH2* hampering either the association of N-WASP to amph2 or the association of the N-WASP–amph2 complex to membranes. How mutations in *DNM2* lead to disruption of amph2 and N-WASP localization and function is currently unknown.

*AMPH2* mRNA splicing is misregulated in skeletal muscle of myotonic dystrophy patients leading to the expression of amph2 lacking the phosphoinositide-binding (PI) domain encoded by exon 11, which is involved in the targeting of AMPH2 to triads (Lee *et al*, 2002; Fugier *et al*, 2011). Amph2 splicing misregulation is associated with the disorganization of triads observed in myotonic dystrophy skeletal muscles. Interestingly, our data suggest that N-WASP is also involved in DM1 pathology since we observed a disruption of the localization of N-WASP on DM1 muscle, as previously reported for amph2 (Fig 8C) (Fugier *et al*, 2011). Therefore, misregulation of N-WASP function might also underlie the impairment of muscle function in myotonic dystrophy.

Finally, we showed that expression of active N-WASP (VCA domain) can revert the defects on nuclear positioning and triad organization caused by downregulation of amph2 or dynamin-2 (Fig 5 and Supplementary Fig S4). Therefore, activation of N-WASP in skeletal muscle might improve muscle function in patients with centronuclear myopathy, myotonic dystrophy, and other multiple muscle disorders characterized by defects in nuclear positioning and triad organization (Cowling *et al*, 2012).

## Materials and Methods

### Antibodies

The following antibodies were used: mouse anti-DHPR (IF) 1:500 from Chemicon; rabbit anti-triadin (TRISK95) (IF) 1:500 is described in (Marty *et al*, 2000); mouse anti-amph2 clone 99D (Upstate) (IF) 1:200; (WB) 1:1,000; (IP) 2 µg, and rabbit anti-amph2 2406 (WB) 1:1,000 is described in (Toussaint *et al*, 2011); rabbit anti-N-WASP from EMC (IF) 1:200 and (WB) 1:1,000 for and mouse anti-N-WASP from Sigma (IF) 1:200; (WB) 1:1,000; (IP) 10 µg; mouse anti-RyR from Sigma (IF) 1:200; mouse anti-GAPDH (WB) 1:5,000 (IF) 1:500 from Ambion; mouse anti-beta-tubulin (WB) 1:1,000 from Sigma; mouse anti-actinin EA53 from Sigma (IF) 1:800; rabbit anti-calnexin from Abcam (WB) 1:2,000. Anti-tubulin (IF) 1:50 (from rat monoclonal anti-α-tubulin (YL1/2)-producing hybridoma cell line, ATCC).

### Plasmids and siRNA

GFP-tagged human amph2-K573X, amph2-K575X, amph2-D151N, and amph2-R154Q were described in (Nicot *et al*, 2007). GFP-tagged N-WASP-FL or VCA (Lommel *et al*, 2001) were a gift from Theresia Stradal. pTdT-VCA has been generated by NheI-BsrGI replacement of GFP with pTdT in GFP-VCA. pcDNA GFP was a gift from Alexis Gautreau. RFP-KASHΔL and RFP-SR-KASH were previously described in (Luxton *et al*, 2010). The following siRNAs were purchased from Life Technologies; *amph2* exon3 siRNA (GGAUCUUCGGACCUAUCUGtt); *N-WASP* siRNAs (#1: GGCUAAUCCACUCUGAGUAtt and #2: GGCUAUUUUUUAGCAAAGAtt); *GAPDH* siRNA (cat # AM4624); *DNM2* siRNA (GCGAAUUGAAGGCUCGGGAtt), *MTM1* siRNA (CGCAUAUCAAACUCAGAAtt), amph2 exon11 siRNA (CGGCUGCGCAGAAAGAAGAtt), *Kif5b* siRNA (TAGACCGGATAAAGGAAGCAG), and *Map7* siRNA (CAGAUUAGAUGUCACCAAUTT).

### Western blotting

Cells were lysed in PBS + 1% SDS and passed through a Qiashredder column (Qiagen) to disrupt DNA. For human muscle, protein extracts were obtained from 200–300 sections from each biopsie and lysed in RIPA buffer.

Protein concentration was measured with a BCA kit according to manufacturer instructions (Pierce). Equal amount of sample were boiled in 30 µl sample buffer and were loaded on 4–12% precast Bis-Tris gel (Invitrogen) and transferred into nitrocellulose membrane using the iBlot apparatus (Invitrogen). Membranes were blocked with blocking buffer (5% non-fat dry milk, 0.1% Tween in TBS). Primary antibodies were incubated overnight in blocking buffer at 4°C. After three washes with TBS-Tween 0.1%, membranes were incubated with HRP-conjugated secondary antibodies (1 h at room temperature). Proteins were visualized using ECL reagent (Pierce).

### PCR

After lysis with RIPA buffer, the sample were divided in two parts, one were precipitated with acidic phenol/chloroform (pH 4.5) for RNA extraction and the other were used for differential centrifugation followed by Western blot as described before. cDNAs were synthesised from 2 to 5 μg of total RNA using Superscript II reverse transcriptase (Invitrogen) and random hexamers. Quantitative PCR amplification of cDNAs was performed on Light Cycler 480 and Light Cycler 24 instruments (Roche) using 58°C as melting temperature. Primers used were DHPRa1S (CACNA1S) (F-GCTACTTTGGAGACCCCTGGAA, R-CAGAAGGAGTGCGAACCCTCCT), N-Wasp (WASL) (F-CAGAGGCACAACTTAAAGACAGAG, R-CTCCTTCCAGGCCAAGTGTAG), MTM1 (F- TGGAAGAATACAGGAGGC, R- TGGAATTCGATTTCGGGAC) BIN1 (F-TCTCCAGAAGCTGGGGAAG, R-TGACTTGATGTCGGGGAACT), DNM2 (F-GAGTTTGACGAGAAGGACTTA, R- GATTAGCTCCTGGATAACCAG), and 18S (F-CGCCGCTAGAGGTGAAATTC, R-TTGGCAAATGCTTTCGCTC).

### Immunoprecipitation

For immunoprecipitation of endogenous N-WASP or amph2, anti-N-WASP mAb or anti-amph2 mAb were cross-linked to magnetic Dynabeads protein G (Invitrogen) using BS3 linker (Fisher Scientific) following manufacturer instructions. Cells were lysed in RIPA buffer, centrifuged at 2,650 *g* for 5 min, and cell lysates were precleared with dynabeads for 30 min at 4°C. Supernatants were incubated with the antibody-coupled Dynabeads for 60 min at 4°C, washed with RIPA buffer, and dynabeads were boiled in 20 µl of sample buffer and analyzed by Western blot. For immunoprecipitation of GFP-tagged proteins, a GFP-Trap system (Chromotek) was used following manufacturer instructions. After thorough washing with the lysis buffer, bound proteins were eluted in sample buffer boiled and analyzed by Western blot.

For immunoprecipitation in muscle, fresh mouse tibialis anterior muscles (8 weeks old) were dissected and homogenized with a dounce homogenizer in ice-cold co-IP buffer (50 mM Tris-Cl, pH 7.5; 100 mM NaCl; 5 mM EDTA; 5 mM EGTA; 1 mM DTT; 0.5% Triton X-100; and 2 mM PMSF) supplemented with 0.05% (w/v) SDS. Lysates were centrifuged at 14,000 *g* at 4°C and precleared with 50 μl of G-sepharose beads (GE Healthcare) and subsequently incubated with antibodies of interest for 12–24 h at 4°C. Protein G-sepharose beads (50 ml) were then added for 4 h at 4°C to capture the immune complexes. Beads were washed four times with co-IP buffer and one time with high stringency co-IP buffer (with 300 mM NaCl). For all experiments, two negative controls consisted of a sample lacking the primary antibody (Beads) and a sample incubated with IgG antibody (IGBMC, Illkirch, France). Resulting immune-bound complexes were eluted in Laemmli buffer and submitted to SDS-PAGE and Western blot analysis.

### GST pull down and *in vitro* transcription/translation

cDNA corresponding to full-length, BAR+PI, BAR (aa 1–255), and SH3 (aa 380–454) sequences of BIN1 (isoform 8, 454 aa) and to full-length sequence of MTM1 were cloned into pENTR1A Gateway entry vector (Invitrogen) and recombined into pDEST15 (N-ter GST fusion) destination vectors. Mutations were introduced in pENTR1A-BIN1 full-length vector using primer-directed PCR mutagenesis. All the constructs were verified by Sanger sequencing. GST fusion proteins were expressed in the *E. coli* strain BL21-Rosetta 2 (Novagen). Recombinant proteins were extracted from bacterial pellets by adding extraction buffer (50 mM Tris-HCl (pH 8.0), 100 mM NaCl, 5 mM EDTA, 1 mM EDTA) supplemented with 1 mg/ml lysosyme, a cocktail of protease inhibitors (Complete EDTA free, Roche) and 1 mM PMSF. After 30 min incubation on ice, detergents were added (0.01% N-laurylsarcosine and 0.5% Triton X-100) and lysates were incubated overnight at 4°C to obtain high solubilization. Then, lysates were centrifuged at 16,000 *g* for 30 min. GST fusion proteins were purified by incubation with glutathione sepharose 4B beads (GE Healthcare) overnight followed by extensive washing with extraction buffer plus 0.5% Triton X-100.

The purified GST fusion proteins coupled to glutathione beads were then incubated overnight with N-WASP translated *in vitro* (TNT Coupled Reticulocyte Lysate Systems, Promega) according to the manufacturer's protocol. Briefly, pEGFP-N-WASP plasmid was incubated with Methionine and TNT T7 quick master mix for 2 h at 30°C. The homogenates were diluted with extraction buffer 50 mM Tris-HCl (pH 8.0), 100 mM NaCl, 5 mM EDTA, 1 mM EDTA) supplemented with a cocktail of protease inhibitors (Complete EDTA free, Roche) and 1 mM PMSF. After washing beads several times with extraction buffer, bound proteins were analyzed by Western blot.

### Muscle homogenate differential centrifugation

Differential centrifugation of muscle from control and ARCNM biopsies homogenates have been used to obtain different fractions. The homogenate was centrifuged at 1,500 *g* for 5 min at 4°C, producing a low-speed pellet rich in myofibrils (1,500 *g* pellet). After washing the low-speed pellet four times in rigor buffer, the low-speed supernatant was centrifuged at 15,000 *g* for 15 min at 4°C, producing a medium-speed pellet rich in organelles, membranes, and other extrasarcomeric structures (15,000 *g* pellet). The resulting medium-speed supernatant was then centrifuged at 150,000 *g* for 15 min at 4°C, producing a high-speed pellet rich in microsomes and macromolecular complexes (150,000 *g* pellet) and a high-speed supernatant containing cytosol.

### Cell culture, reagents, and TA muscle fiber isolation

All procedures using animals were approved by the Institutional ethics committee and followed the guidelines of the National Research Council Guide for the care and use of laboratory animals. Primary myoblasts from WT or H2B-GFP (The Jackson Laboratories, STOCK Tg(HIST1H2BB/EGFP)1Pa/J) newborn mice were prepared using a protocol adapted from (De Palma *et al*, 2010). H2B-GFP colony was maintained in hemozygous conditions and WT or H2B-GFP transgenic pups were obtained from the same litter. After hind limb muscles isolation, muscles were minced and digested for 1.5 h in PBS containing 0.5 mg/ml collagenase (Sigma) and 3.5 mg/ml dispase (Gibco). Cell suspension was filtered through a 40-µm cell strainer and preplated in DMED + 10%FBS (Gibco), to discard the majority of fibroblasts and contaminating cells, for 3 h. Non-adherent-myogenic cells were collected and plated in IMDM (Gibco) + 20% FBS + 1% Chick Embryo Extract (MP Biomedical) onto 1:100 Matrigel Reduced Factor (BD) in IMDM-coated fluorodishes. Differentiation was triggered by medium switch in IMDM + 2% horse serum, and 24 h later, a thick layer of matrigel (1:3 in IMDM) was added. Myotubes were treated with 80 μg/ml of agrin, and the medium was changed every 2 days (see scheme in Supplementary Fig S1).

Latrunculin B (EMD Millipore) and nocodazole (Sigma) were used at 10 and 75 nM, respectively, starting at day 1 or day 5 after agrin addition (see scheme in Supplementary Fig S1A). Medium was changed every 2 days supplemented with the drugs. C2C12 cell line was cultured as described (De Palma *et al*, 2010) in DMEM + 10% FBS (Gibco).

TA single fibers were isolated as described (Rosenblatt *et al*, 1995). TA muscle was explanted from 8- to 12-week-old male or female CD1 mice and then digested in DMEM containing 0.2% type I collagenase (Sigma) for 2 h at 37°C. Mechanical dissociation of fibers was performed using a thin pasteur pipette and followed under a transilluminating-fluorescent stereomicroscope.

### Immunofluorescence and immunohistochemistry

The same protocol for either *in vitro* myofibers or isolated muscle fibers was used. Briefly, fluorodishes or fibers were fixed in 4% paraformaldehyde (PFA) for 10 min, permeabilized with Triton X-100 (0.5% in PBS), and aspecific sites were blocked with BSA 1% and goat serum 10% for 30 min. Primary antibodies were added overnight at 4°C in 0.1% saponin and 1% BSA in PBS. Fluorodishes or fibers were washes three times and then incubated with secondary antibodies together with DAPI for 60 min.

### Peripheral nuclei quantification

Myofibers were stained for DHPR1 and DAPI and images in Z-stacks with 0.5-μm interval were acquired with a Leica SPE confocal microscope with a 63× 1.3 NA Apo objective. Nuclei extruding the myofiber periphery and positively stained for DHPR were scored as peripheral. A minimum of 150 nuclei per condition were counted in at least three individual experiments.

### Triad quantification

Myofibers were stained for DHPR1, triadin, and DAPI and images in Z-stacks with 0.5-μm interval were acquired with a Leica SPE confocal microscope with a 63× 1.3 NA Apo objective. Myofibers having more than 50% of triads organized, where DHPR and triadin were transversally overlapping, were scored as positive. A minimum of 150 myofibers per condition were counted in at least three individual experiments.

### Fiber thickness quantification

For fiber thickness quantification, myofibers stained for caveolin-3 to visualize plasma membrane were observed with a Leica SPE confocal microscope with a 40× 1.3 NA Apo objective. Average of three measurements per myofiber was performed on 100 myofibers analyzed per each condition in at least three individual experiments.

### Human biopsies

All patients or their families have agreed and signed the consent for human sample use according to French and Japanese legislation. Experiments were conformed to the principles set out in the WMA Declaration of Helsinki and the NIH Belmont Report. Biopsies used for protein homogenates to perform Western blot and immunoprecipitation experiments were obtained from two different healthy donors (46 and 27 years old) as control. Both XLCNM patients were 1-month-old with MTM1 c(455-49)-445-4)del and MTM1-R421insFIG, respectively. ADCNM patients were 16 (E368K mutation) and 3 (R456W mutation) years old, respectively. For ARCNM patients, we used K575X and D151N mutated patient biopsies, previously described in (Toussaint *et al*, 2011). All biopsies were frozen in liquid nitrogen cooled isopentane (SIGMA) and kept at −80°C until use. A biopsy from 35-year-old DM1 patient [described in (Fugier *et al*, 2011)] was also used. Age-matched healthy donor biopsies were used as control for each myopathy.

### Cryosectioning and immunofluorescence microscopy

All procedures using animals were approved by the Institutional ethics committee and followed the guidelines of the National Research Council Guide for the care and use of laboratory animals. For cryosectioning, the TA muscles were isolated from 8- to 12-week-old male or female CD1 mice and frozen in isopentane precooled with liquid nitrogen. Cryosections (10 μm thick) were fixed in 4% PFA for 30 min. Permeabilization was performed in cooled methanol, and aspecific sites were blocked in 5% BSA. For human biopsies, sections were prepared as described in (Fugier *et al*, 2011; Toussaint *et al*, 2011). Sections were then incubated with primary antibodies overnight in PBS containing 4% BSA, rinsed, and incubated with fluorescent secondary antibodies and DAPI for 1 h at RT.

### Microscopy

Live imaging was performed using an incubator to maintain cultures at 37°C and 5% CO_2_ (Okolab) and ×20 0.3 NA PL Fluo dry objective. Epi-fluorescence images were acquired using a Nikon Ti microscope equipped with a CoolSNAP HQ2 camera (Roper Scientific), an XY-motorized stage (Nikon), driven by Metamorph (Molecular Devices). Multipositioning images were stitched with Metamorph (Molecular Devices). Confocal images were acquired using Leica SPE confocal microscope with a ×63 1.3 NA Apo objective.

Electron microscopy was performed on cultured myofibers 10 days after agrin addition. Myofibers were fixed with 2% glutaraldehyde, 2% PFA in 0.1 M phosphate buffer (pH 7.4), and post-fixed with 2% OsO_4_ in 0.1 M phosphate buffer for 30 min at 4°C. Myofibers were then dehydrated at 4°C in acetone and stained with 1% uranyl acetate in 70% (vol/vol) acetone, before Epon resin embedding. Thin (70 nm) sections were stained with uranyl acetate and lead citrate, observed using a Philips CM120 electron microscope (Philips Electronics NV) and photographed with a digital SIS Morada camera.

### Transfection and electroporation

Cells were transfected with siRNA (20 nM) using RNAiMAX, cDNA using Lipofectamine-LTX Plus reagent or both using Lipofectamine 2000 following manufacturer instructions (Invitrogen). Primary myoblasts were transfected 6 h prior to differentiation, to allow protein silencing or overexpression effective after differentiation (see scheme in Supplementary Fig S1).

For *in vivo* muscle transfection using electroporation, we used procedures approved by the Institutional ethics committee and followed the guidelines of the National Research Council Guide for the care and use of laboratory animals. siRNAs were electroporated together with p-CITO-tdTomato (derived from pCig by substituting nlsGFP with tdTomato cDNA) as fluorescent reporter of transfection. 20 μg of each plasmid DNA or 40 μg of siRNA were dissolved in 40 μl of PBS and injected into the TA muscle of 8- to 12-week-old CD1 mice (male or female) using a 27-gauge needle centered between a pair of needle electrodes (Nepa Gene). Immediately after the plasmid injection, six pulses of 60-ms duration at a voltage of 200 V/cm were administered with a square pulse electroporator system CUY21SC (Nepa Gene). Muscles were retrieved 10 days after electroporation to either cryosectioning or muscle fiber isolation.

### Calcium assay

Calcium assay was performed on fully differentiated myofibers at day 10 after agrin addition. Fluo-4 was prepared according to manufacturer instructions (Life Technologies ref# F10471). The myofibers were bathed in the Fluo-4 solution at 37°C for 30 min and were then incubated at RT for another 30 min. The myofibers were subsequently imaged using an argon laser with peak wavelength at 488 nm, on an Epi-fluorescence Nikon Ti microscope driven by Metamorph (Molecular Devices) with image acquisition every 6 s. Caffeine (50 mM) or KCl (50 mM) was added after 100 s. Average of fluorescent intensity of Fluo-4 was performed on 30 myofibers analyzed per each condition in at least three individual experiments.

### Statistics

Statistical analysis was performed with Prism (version 5.0 of GraphPad Software Inc.). Pairwise comparisons were made with Student's *t*-test. In peripheral nuclei positioning analysis and in fiber thickness analysis in myofibers, Student's *t*-tests were performed between *GAPDH* siRNA and experimental condition. For biochemical experiments using human samples, statistical analysis was performed using the Mann–Whitney *U*-test or the unpaired Student's *t*-test and multiple statistical comparisons between samples were performed by one-way analysis of variance followed by a Bonferroni's *t*-test *post hoc* correction to obtain a better evaluation of the variability between samples from the same group and samples from each compared group and statistical significance was set at *P ≤ 0.05, **P < 0.01, and ***P < 0.001. The prism program (version 5.0, GraphPad software Inc.) was used. The distribution of data points is expressed as mean ± s.e.m. from three independent experiments.

## For more information

Patient associations:

Myotubular trust: http://www.myotubulartrust.com

French Muscular Dystrophy Association: http://www.afm-telethon.com

The paper explained Problem Centronuclear myopathies (CNM) and myotonic dystrophy have common pathological features such as centrally positioned nuclei and defects in triad organization in skeletal muscle. Both these disorders can be caused by alterations in *BIN1/AMPH2* gene. Results In our work, we describe a novel *in vitro* system to study muscle disorders. We found a novel protein that is regulated by BIN1/amphiphysin-2, named N-WASP, that is important for nuclear positioning and triad organization. We also found that N-WASP distribution is disrupted in skeletal muscle fibers from CNM and myotonic dystrophy patients. Furthermore, we show that activation of N-WASP can revert the pathological features of CNM *in vitro*. Impact Our work suggests that activation of N-WASP can be a new therapeutic approach for the treatment of CNM and other muscles disorders. Furthermore, the *in vitro* system that we describe in this work provides a platform to screen for potential drugs to ameliorate or treat muscle disorders.

## References

[b1] Al-Qusairi L, Laporte J (2011). T-tubule biogenesis and triad formation in skeletal muscle and implication in human diseases. Skelet Muscle.

[b2] Al-Qusairi L, Weiss N, Toussaint A, Berbey C, Messaddeq N, Kretz C, Sanoudou D, Beggs AH, Allard B, Mandel J-L (2009). T-tubule disorganization and defective excitation-contraction coupling in muscle fibers lacking myotubularin lipid phosphatase. Proc Natl Acad Sci USA.

[b3] Biancalana V, Caron O, Gallati S, Baas F, Kress W, Novelli G, D'Apice MR, Lagier-Tourenne C, Buj-Bello A, Romero NB (2003). Characterisation of mutations in 77 patients with X-linked myotubular myopathy, including a family with a very mild phenotype. Hum Genet.

[b4] Bitoun M, Maugenre S, Jeannet P-Y, Lacène E, Ferrer X, Laforêt P, Martin J-J, Laporte J, Lochmüller H, Beggs AH (2005). Mutations in dynamin 2 cause dominant centronuclear myopathy. Nat Genet.

[b5] Blau HM, Pavlath GK, Hardeman EC, Chiu CP, Silberstein L, Webster SG, Miller SC, Webster C (1985). Plasticity of the differentiated state. Science.

[b6] Bruusgaard JC, Liestøl K, Ekmark M, Kollstad K, Gundersen K (2003). Number and spatial distribution of nuclei in the muscle fibres of normal mice studied in vivo. J Physiol.

[b7] Bugyi B, Didry D, Carlier M-F (2010). How tropomyosin regulates lamellipodial actin-based motility: a combined biochemical and reconstituted motility approach. EMBO J.

[b8] Butler MH, David C, Ochoa GC, Freyberg Z, Daniell L, Grabs D, Cremona O, De Camilli P (1997). Amphiphysin II (SH3P9; BIN1), a member of the amphiphysin/Rvs family, is concentrated in the cortical cytomatrix of axon initial segments and nodes of ranvier in brain and around T tubules in skeletal muscle. J Cell Biol.

[b9] Cadot B, Gache V, Vasyutina E, Falcone S, Birchmeier C, Gomes ER (2012). Nuclear movement during myotube formation is microtubule and dynein dependent and is regulated by Cdc42, Par6 and Par3. EMBO Rep.

[b10] Cooper ST, Maxwell AL, Kizana E, Ghoddusi M, Hardeman EC, Alexander IE, Allen DG, North KN (2004). C2C12 co-culture on a fibroblast substratum enables sustained survival of contractile, highly differentiated myotubes with peripheral nuclei and adult fast myosin expression. Cell Motil Cytoskeleton.

[b11] Cowling BS, Toussaint A, Muller J, Laporte J (2012). Defective membrane remodeling in neuromuscular diseases: insights from animal models. PLoS Genet.

[b12] Cusimano V, Pampinella F, Giacomello E, Sorrentino V (2009). Assembly and dynamics of proteins of the longitudinal and junctional sarcoplasmic reticulum in skeletal muscle cells. Proc Natl Acad Sci USA.

[b13] Davis RL, Weintraub H, Lassar AB (1987). Expression of a single transfected cDNA converts fibroblasts to myoblasts. Cell.

[b14] De Palma C, Falcone S, Pisoni S, Cipolat S, Panzeri C, Pambianco S, Pisconti A, Allevi R, Bassi MT, Cossu G (2010). Nitric oxide inhibition of Drp1-mediated mitochondrial fission is critical for myogenic differentiation. Cell Death Differ.

[b16] Dowling JJ, Vreede AP, Low SE, Gibbs EM, Kuwada JY, Bonnemann CG, Feldman EL (2009). Loss of myotubularin function results in T-tubule disorganization in zebrafish and human myotubular myopathy. PLoS Genet.

[b17] Flucher BE (1992). Structural analysis of muscle development: transverse tubules, sarcoplasmic reticulum, and the triad. Dev Biol.

[b18] Flucher BE, Andrews SB, Daniels MP (1994). Molecular organization of transverse tubule/sarcoplasmic reticulum junctions during development of excitation-contraction coupling in skeletal muscle. Mol Biol Cell.

[b19] Flucher BE, Phillips JL, Powell JA (1991). Dihydropyridine receptor alpha subunits in normal and dysgenic muscle in vitro: expression of alpha 1 is required for proper targeting and distribution of alpha 2. J Cell Biol.

[b20] Franzini-Armstrong C, Engel A, Banker BO (1986). The sarcoplasmic reticulum and the transverse tubules. Myology.

[b21] Friesen H, Humphries C, Ho Y, Schub O, Colwill K, Andrews B (2006). Characterization of the yeast amphiphysins Rvs161p and Rvs167p reveals roles for the Rvs heterodimer in vivo. Mol Biol Cell.

[b22] Fugier C, Klein AF, Hammer C, Vassilopoulos S, Ivarsson Y, Toussaint A, Tosch V, Vignaud A, Ferry A, Messaddeq N (2011). Misregulated alternative splicing of BIN1 is associated with T tubule alterations and muscle weakness in myotonic dystrophy. Nat Med.

[b23] Gokhin DS, Fowler VM (2011). Cytoplasmic gamma-actin and tropomodulin isoforms link to the sarcoplasmic reticulum in skeletal muscle fibers. J Cell Biol.

[b24] Grady RM, Starr DA, Ackerman GL, Sanes JR, Han M (2005). Syne proteins anchor muscle nuclei at the neuromuscular junction. Proc Natl Acad Sci USA.

[b25] Gruenbaum-Cohen Y, Harel I, Umansky K-B, Tzahor E, Snapper SB, Shilo B-Z, Schejter ED (2012). The actin regulator N-WASp is required for muscle-cell fusion in mice. Proc Natl Acad Sci USA.

[b26] Hadjantonakis A-K, Papaioannou VE (2004). Dynamic in vivo imaging and cell tracking using a histone fluorescent protein fusion in mice. BMC Biotechnol.

[b27] Hong T, Yang H, Zhang S-S, Cho HC, Kalashnikova M, Sun B, Zhang H, Bhargava A, Grabe M, Olgin J (2014). Cardiac BIN1 folds T-tubule membrane, controlling ion flux and limiting arrhythmia. Nat Med.

[b28] Ito K, Komazaki S, Sasamoto K, Yoshida M, Nishi M, Kitamura K, Takeshima H (2001). Deficiency of triad junction and contraction in mutant skeletal muscle lacking junctophilin type 1. J Cell Biol.

[b29] Kee AJ, Gunning PW, Hardeman EC (2009). Diverse roles of the actin cytoskeleton in striated muscle. J Muscle Res Cell Motil.

[b30] Kee AJ, Schevzov G, Nair-Shalliker V, Robinson CS, Vrhovski B, Ghoddusi M, Qiu MR, Lin JJ-C, Weinberger R, Gunning PW (2004). Sorting of a nonmuscle tropomyosin to a novel cytoskeletal compartment in skeletal muscle results in muscular dystrophy. J Cell Biol.

[b33] Laporte J, Hu LJ, Kretz C, Mandel JL, Kioschis P, Coy JF, Klauck SM, Poustka A, Dahl N (1996). A gene mutated in X-linked myotubular myopathy defines a new putative tyrosine phosphatase family conserved in yeast. Nat Genet.

[b34] Lee E, Marcucci M, Daniell L, Pypaert M, Weisz OA, Ochoa G-C, Farsad K, Wenk MR, De Camilli P (2002). Amphiphysin 2 (Bin1) and T-tubule biogenesis in muscle. Science.

[b350] Lommel S, Benesch S, Rottner K, Franz T, Wehland J, Kühn R (2001). Actin pedestal formation by enteropathogenic *Escherichia coli* and intracellular motility of *Shigella flexneri* are abolished in N-WASP-defective cells. EMBO Rep.

[b35] Luxton GWG, Gomes ER, Folker ES, Vintinner E, Gundersen GG (2010). Linear arrays of nuclear envelope proteins harness retrograde actin flow for nuclear movement. Science.

[b36] Madania A, Dumoulin P, Grava S, Kitamoto H, Schärer-Brodbeck C, Soulard A, Moreau V, Winsor B (1999). The *Saccharomyces cerevisiae* homologue of human Wiskott-Aldrich syndrome protein Las17p interacts with the Arp2/3 complex. Mol Biol Cell.

[b37] Marty I, Thevenon D, Scotto C, Groh S, Sainnier S, Robert M, Grunwald D, Villaz M (2000). Cloning and characterization of a new isoform of skeletal muscle triadin. J Biol Chem.

[b39] Metzger T, Gache V, Xu M, Cadot B, Folker ES, Richardson BE, Gomes ER, Baylies MK (2012). MAP and kinesin-dependent nuclear positioning is required for skeletal muscle function. Nature.

[b40] Nicot A-S, Toussaint A, Tosch V, Kretz C, Wallgren-Pettersson C, Iwarsson E, Kingston H, Garnier J-M, Biancalana V, Oldfors A (2007). Mutations in amphiphysin 2 (BIN1) disrupt interaction with dynamin 2 and cause autosomal recessive centronuclear myopathy. Nat Genet.

[b41] Padrick SB, Rosen MK (2010). Physical mechanisms of signal integration by WASP family proteins. Annu Rev Biochem.

[b42] Parton RG, Way M, Zorzi N, Stang E (1997). Caveolin-3 associates with developing T-tubules during muscle differentiation. J Cell Biol.

[b43] Pierson CR, Tomczak K, Agrawal P, Moghadaszadeh B, Beggs AH (2005). X-linked myotubular and centronuclear myopathies. J Neuropathol Exp Neurol.

[b44] Powell JA, Petherbridge L, Flucher BE (1996). Formation of triads without the dihydropyridine receptor alpha subunits in cell lines from dysgenic skeletal muscle. J Cell Biol.

[b45] Prehoda KE, Scott JA, Mullins RD, Lim WA (2000). Integration of multiple signals through cooperative regulation of the N-WASP-Arp2/3 complex. Science.

[b46] Prokic I, Cowling BS, Laporte J (2014). Amphiphysin 2 (BIN1) in physiology and diseases. J Mol Med.

[b47] Razzaq A, Robinson IM, McMahon HT, Skepper JN, Su Y, Zelhof AC, Jackson AP, Gay NJ, O'Kane CJ (2001). Amphiphysin is necessary for organization of the excitation-contraction coupling machinery of muscles, but not for synaptic vesicle endocytosis in Drosophila. Genes Dev.

[b48] Rohatgi R, Ma L, Miki H, Lopez M, Kirchhausen T, Takenawa T, Kirschner MW (1999). The interaction between N-WASP and the Arp2/3 complex links Cdc42-dependent signals to actin assembly. Cell.

[b49] Rosenblatt JD, Lunt AI, Parry DJ, Partridge TA (1995). Culturing satellite cells from living single muscle fiber explants. In Vitro Cell Dev Biol Anim.

[b51] Salazar MA, Kwiatkowski AV, Pellegrini L, Cestra G, Butler MH, Rossman KL, Serna DM, Sondek J, Gertler FB, Camilli PD (2003). Tuba, a novel protein containing Bin/Amphiphysin/Rvs and Dbl homology domains, links dynamin to regulation of the actin cytoskeleton. J Biol Chem.

[b52] Sonnemann KJ, Fitzsimons DP, Patel JR, Liu Y, Schneider MF, Moss RL, Ervasti JM (2006). Cytoplasmic gamma-actin is not required for skeletal muscle development but its absence leads to a progressive myopathy. Dev Cell.

[b53] Starr DA, Fridolfsson HN (2010). Interactions between nuclei and the cytoskeleton are mediated by SUN-KASH nuclear-envelope bridges. Annu Rev Cell Dev Biol.

[b54] Suetsugu S, Gautreau A (2012). Synergistic BAR-NPF interactions in actin-driven membrane remodeling. Trends Cell Biol.

[b55] Takano K, Watanabe-Takano H, Suetsugu S, Kurita S, Tsujita K, Kimura S, Karatsu T, Takenawa T, Endo T (2010). Nebulin and N-WASP cooperate to cause IGF-1-induced sarcomeric actin filament formation. Science.

[b56] Takekura H, Flucher BE, Franzini-Armstrong C (2001). Sequential docking, molecular differentiation, and positioning of T-Tubule/SR junctions in developing mouse skeletal muscle. DevBiol.

[b57] Taneike M, Mizote I, Morita T, Watanabe T, Hikoso S, Yamaguchi O, Takeda T, Oka T, Tamai T, Oyabu J (2011). Calpain protects the heart from hemodynamic stress. J Biol Chem.

[b58] Tjondrokoesoemo A, Park KH, Ferrante C, Komazaki S, Lesniak S, Brotto M, Ko J-K, Zhou J, Weisleder N, Ma J (2011). Disrupted membrane structure and intracellular Ca^2+^ signaling in adult skeletal muscle with acute knockdown of Bin1. PLoS ONE.

[b59] Toussaint A, Cowling BS, Hnia K, Mohr M, Oldfors A, Schwab Y, Yis U, Maisonobe T, Stojkovic T, Wallgren-Pettersson C (2011). Defects in amphiphysin 2 (BIN1) and triads in several forms of centronuclear myopathies. Acta Neuropathol.

[b60] Vasli N, Laugel V, Bohm J, Lannes B, Biancalana V, Laporte J (2012). Myotubular myopathy caused by multiple abnormal splicing variants in the MTM1 RNA in a patient with a mild phenotype. Eur J Hum Genet.

[b61] Vlahovich N, Kee AJ, Van der Poel C, Kettle E, Hernandez-Deviez D, Lucas C, Lynch GS, Parton RG, Gunning PW, Hardeman EC (2009). Cytoskeletal tropomyosin Tm5NM1 is required for normal excitation-contraction coupling in skeletal muscle. Mol Biol Cell.

[b62] Wang Z, Cui J, Wong WM, Li X, Xue W, Lin R, Wang J, Wang P, Tanner JA, Cheah KSE (2013). Kif5b controls the localization of myofibril components for their assembly and linkage to the myotendinous junctions. Development.

[b63] Wu X, Yoo Y, Okuhama NN, Tucker PW, Liu G, Guan J-L (2006). Regulation of RNA-polymerase-II-dependent transcription by N-WASP and its nuclear-binding partners. Nat Cell Biol.

[b64] Yamada H, Padilla-Parra S, Park S-J, Itoh T, Chaineau M, Monaldi I, Cremona O, Benfenati F, De Camilli P, Coppey-Moisan M (2009). Dynamic interaction of amphiphysin with N-WASP regulates actin assembly. J Biol Chem.

[b65] Zalk R, Lehnart SE, Marks AR (2007). Modulation of the ryanodine receptor and intracellular calcium. Annu Rev Biochem.

[b66] Zelhof AC, Hardy RW (2004). WASp is required for the correct temporal morphogenesis of rhabdomere microvilli. J Cell Biol.

[b67] Zhang X, Xu R, Zhu B, Yang X, Ding X, Duan S, Xu T, Zhuang Y, Han M (2007). Syne-1 and Syne-2 play crucial roles in myonuclear anchorage and motor neuron innervation. Development.

[b68] Zheng Z, Wang Z-M, Delbono O (2002). Charge movement and transcription regulation of L-type calcium channel alpha(1S) in skeletal muscle cells. J Physiol (Lond).

