# Supplementary material for: N-WASP is required for Amphiphysin-2/BIN1-dependent nuclear positioning and triad organization in skeletal muscle and is involved in the pathophysiology of centronuclear myopathy
Source: EMBO Mol Med. 2014 Sep 29;6(11):1455–75. doi: 10.15252/emmm.201404436 (PMC4237471; doi:10.15252/emmm.201404436)
Supplement: Supplementary file 1 [file emmm0006-1455-sd1.pdf]

EMBO Molecular Medicine

N-WASP is required for Amphiphysin-2/BIN1 dependent nuclear positioning and triad organization in skeletal muscle and is involved in the pathophysiology of centronuclear myopathy

Sestina Falcone, William Roman, Karim Hnia, Vincent Gache, Nathalie Didier, Jeanne Lainné, Frederic Aurade, Isabelle Marty, Ichizo Nishino, Nicolas Charlet-Berguerand,Norma Romero, Giovanna Marazzi, David Sassoon, Jocelyn Laporte, Edgar R. Gomes

Corresponding authors: Edgar Gomes and Sestina Falcone, Université Pierre et Marie Curie Paris V,

Review timeline:

	Submission date:	15 July 2014
	Accepted:	26 August 2014

Editor: Céline Carret

Transaction Report:

(Please note that the manuscript was previously reviewed at another journal and the reports were taken into account in the decision making process at EMBO Molecular Medicine. Since the original reviews are not subject to EMBO's transparent review process policy, the reports and author response cannot be published)
