# Supplementary figures and images for: N-WASP is required for Amphiphysin-2/BIN1-dependent nuclear positioning and triad organization in skeletal muscle and is involved in the pathophysiology of centronuclear myopathy
Source: EMBO Mol Med. 2014 Sep 29;6(11):1455–75. doi: 10.15252/emmm.201404436 (PMC4237471; doi:10.15252/emmm.201404436)

Figure 3

Figure 3A

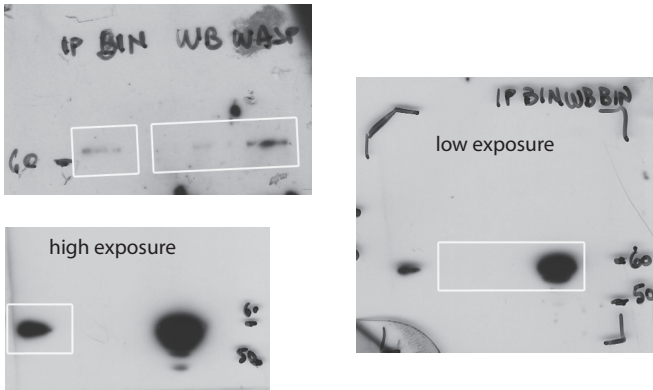

Figure 3F

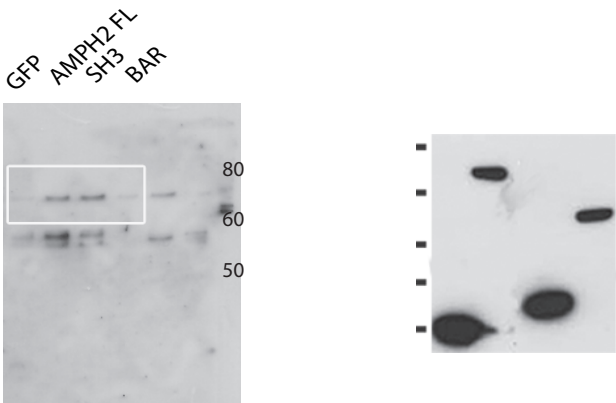

Figure 3B

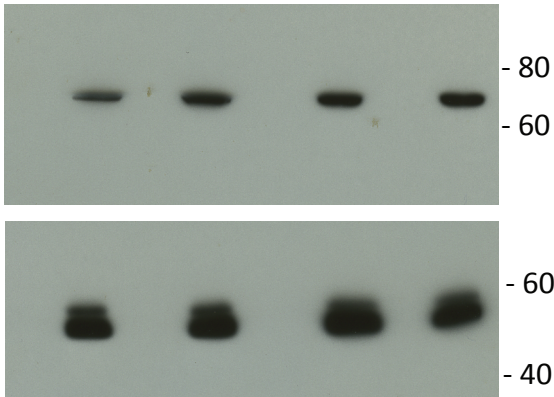

Figure 3G

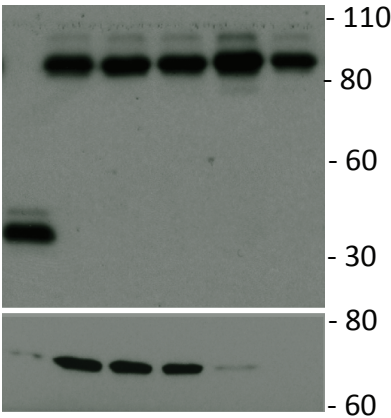

Figure 3C

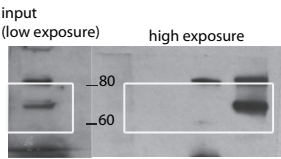

Figure 3H

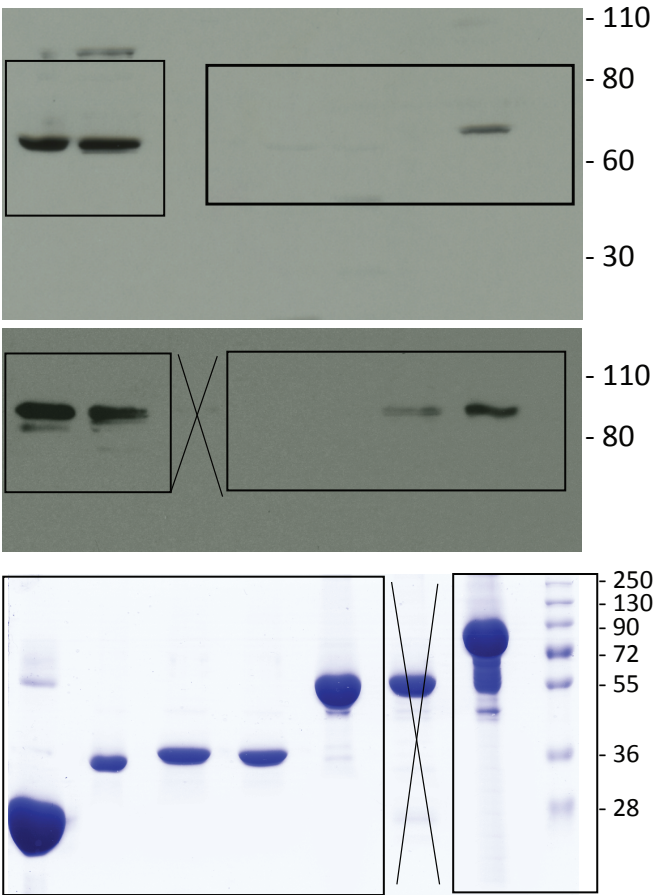

Supplement: Supplementary file 3 [file emmm0006-1455-sd3.pdf]

Figure 5D

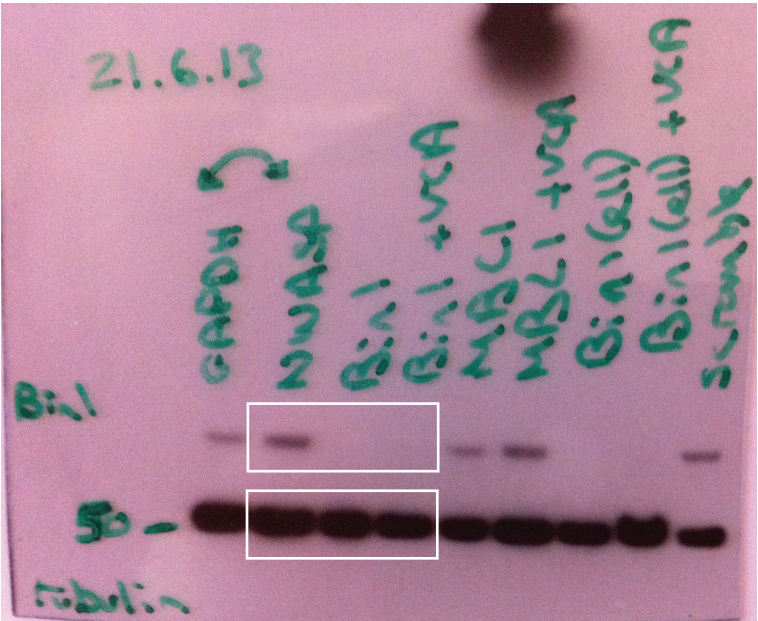

Supplement: Supplementary file 4 [file emmm0006-1455-sd4.pdf]
