# Supplementary material for: N-WASP is required for Amphiphysin-2/BIN1-dependent nuclear positioning and triad organization in skeletal muscle and is involved in the pathophysiology of centronuclear myopathy
Source: EMBO Mol Med. 2014 Sep 29;6(11):1455–75. doi: 10.15252/emmm.201404436 (PMC4237471; doi:10.15252/emmm.201404436)
Supplement: Supplementary file 5 [file emmm0006-1455-sd5.pdf]

TABLE OF CONTENTS

SUPPLEMENTARY LEGENDS .... 2 - 11

MOVIE S1 2
FIGURE S1 LEGEND 3
FIGURE S2 LEGEND 4
FIGURE S3 LEGEND 5
FIGURE S4 LEGEND 6
FIGURE S5 LEGEND 7
FIGURE S6 LEGEND 8
FIGURE S7 LEGEND 9
FIGURE S8 LEGEND 10
FIGURE S9 LEGEND 11

FIGURES .... 12 - 20

FIGURE S1 12
FIGURE S2 13
FIGURE S3 14
FIGURE S4 15
FIGURE S5 16
FIGURE S6 17
FIGURE S7 18
FIGURE S8 19
FIGURE S9 20

Supplementary legends:

Movie S1:
Representative time-lapse phase contrast movie of H2B-GFP primary myotubes, recorded from day 1, after agrin addition, until day 10, showing nuclear movement during myofiber maturation.

Figure S1: *In vitro* myofiber maturation

A) Schematic representation of *in vitro* model protocol, showing the timeline for plasmid and siRNA transfection, differentiation and matrigel embedding and treatments.
B) Representative immunofluorescence image of H2B-GFP primary myofibers treated with agrin for 10 days and immunostained for F-actin (red), DHPR (green) and DAPI (Blue). Arrows indicate mononucleated myoblasts and arrowheads indicate peripheral nuclei in myofibers. Bar, 15 μm.
C) Representative immunofluorescence image of WT primary myofiber trated with agrin for 5 days and immunostained for α-Actinin (red) and F-Actin (green). Bar, 15 μm.
D) Representative immunofluorescence image of WT primary myofiber, transfected with *GAPDH* siRNA, treated with agrin for 10 days and immunostained for β-tubulin (red), and DAPI (Blue). Bar, 15 μm.
E) Representative immunofluorescence image of WT primary myofiber, transfected with *Amph2* siRNA, treated with agrin for 10 days and immunostained for β-Tubulin (red), and DAPI (Blue). Bar, 15 μm.

F) Western Blot with Calnexin2 (Clx2) and β-Tubulin antibodies of myofibers untransfected or transfected with *GAPDH* siRNA or, *Amph2* siRNA or *N-WASP* siRNA and treated with agrin for 10 days.

G) Histograms of relative expression of β-Tubulin showed in F. Error bars, s.e.m.

Figure S2: Amph2, dynamin-2 and myotubularin depletion in cultured primary myofibers and fusion index.

A) Western Blot with GAPDH, Amph2 and β-Tubulin antibodies of myofibers untransfected or transfected with *GAPDH* siRNA or *Amph2*- siRNA and treated with agrin for 10 days.

B-C) Histograms of relative expression of GAPDH (B) or Amph2 (C) in myofibers untransfected or transfected with and *GAPDH* siRNA or *Amph2* siRNA. Error bars, s.e.m. p values from t-test.

D) Western Blot with GAPDH, DNM2 and β-Tubulin antibodies of myofibers untransfected or transfected with *GAPDH* siRNA or *Dnm2*- siRNA and treated with agrin for 10 days.

E - F) Histograms of relative expression of GAPDH (E) or Dnm2 (F) in myofibers untransfected or transfected with and *GAPDH* siRNA or *Dnm2* siRNA. Error bars, s.e.m. p values from t-test.

G) Western Blot with GAPDH, Mtm1 and β-Tubulin antibodies of myofibers untransfected or transfected with *GAPDH* siRNA or *Mtm1* siRNA and treated with agrin for 10 days.

H- I) Histograms of relative expression of GAPDH (H) or Mtm1 (I) in myofibers untransfected or transfected with and *GAPDH* siRNA or *Mtm1* siRNA. Error bars, s.e.m. p values from t-test.

J) Representative immunofluorescence images of WT primary myofibers transfected with *GAPDH* siRNA, treated with agrin for 10 days, immunostained for DHPR (red), triadin (green) and DAPI (blue).. Bar, 15 µm .

K) Representative immunofluorescence images of WT primary myofibers transfected with *Dnm2* siRNA, treated with agrin for 10 days, immunostained for DHPR (red), triadin (green) and DAPI (blue). Bars, 15 µm.

L) Representative immunofluorescence images of WT primary myofibers transfected with *Mtm1* siRNA, treated with agrin for 10 days, immunostained for DHPR (red), triadin (green) and DAPI (blue). Bars, 15 µm.

M) Quantification of peripheral nuclei of myofibers transfected with *GAPDH* siRNA, *Mtm1* siRNA or *Dnm2* siRNA and GFP (light blue) or GFP-VCA (blue), and treated with agrin for 10 days. Error bars, s.e.m. p values from t-test (*GAPDH* siRNA vs *Mtm1* or *Dnm2* siRNA).

N) Quantification of the percentage of transversal triads in myofibers transfected with *GAPDH* siRNA, *Mtm1* siRNA or *Dnm2* siRNA and with GFP(pink fill) or GFP-VCA (red fill) and treated with agrin for 10 days Error bars, s.e.m. p values from t-test (*GAPDH* siRNA vs *Mtm1* or *Dnm2* siRNA).

O) Quantification of myofiber thickness in myofibers transfected with *GAPDH* siRNA, *Mtm1* siRNA or *Dnm2* siRNA and treated with agrin for 10 days. Error bars, s.e.m. p values from t-test (*GAPDH* siRNA vs *Mtm1* or *Dnm2* siRNA).

P) Histogram of the average number of nuclei/myotubes (fusion index) in myofibers untransfected or transfected with *GAPDH* siRNA, *Mtm1* siRNA or *Dnm2* siRNA and treated with agrin for 10 days . Error bars, s.e.m.

Figure S3: Calcium responses in *Amph2*-depleted myofibers. Effects of *N-WASP* depletion and Amph2 mutations in cultured primary myofibers

A-B) Time course of Fluo-4 fluorescence intensity (F/F0) in steady state and after KCl (A) or Caffeine (B) stimulation of WT primary myofibers transfected with *GAPDH* siRNA (blue line) or *Amph2* siRNA (red line), treated with agrin for 10 days.

C) Images from a representative time-lapse calcium imaging of Fluo-4 fluorescence in steady state (0 and 60 sec) and after Caffeine stimulation (arrow) of WT primary myofibers transfected with *GAPDH* siRNA (top) or *Amph2* siRNA (bottom), treated with agrin for 10 days.

D-E) Representative immunofluorescence images of WT primary myofibers expressing GFP-Amph2 R154Q (D) or GFP-Amph2 K575X (E), treated with agrin for 10 days and immunostained for DHPR (red), triadin (green) and DAPI (blue). Bars, 15 µm.

F) Quantification of peripheral nuclei in myofibers expressing GFP, GFP-Amph2 R154Q or GFP-Amph2 K575X and treated with agrin for 10 days. Error bars, s.e.m. p values from t-test (GFP vs GFP-Amph2 R154Q or GFP-Amph2 K575X condition).

G) Quantification of transversal triads in myofibers expressing GFP, GFP-Amph2 R154Q or GFP-Amph2 K575X and treated with agrin for 10 days. Error bars, s.e.m. p values from t-test (GFP vs GFP-Amph2 R154Q or GFP-Amph2 K575X condition).

H) Quantification of myofiber thickness in myofibers expressing GFP or Amph2 GFP R154Q or Amph2 GFP K575X myofibers and treated with agrin for 10 days. Error bars, s.e.m. p values from t-test (GFP vs GFP-Amph2 R154Q or GFP-Amph2 K575X condition).

I) Western blot with N-WASP and β-Tubulin antibodies of myofibers transfected with *GAPDH*-siRNA and *N-WASP* siRNA and treated with agrin for 10 days.

J) Histogram of the relative expression of N-WASP in myofibers transfected with *GAPDH* siRNA and *N-WASP* siRNA and treated with agrin for 10 days. Error bars, s.e.m. p values from t-test.

Fig S4: Specific role of Amph2 in CNM phenotype and rescue by constitutively active N-WASP

A) Western blot with Amph2, GFP and β-Tubulin antibodies of myofibers transfected with *GAPDH* siRNA, *Amph2* ex11 siRNA or *Amph2* ex11 siRNA and VCA-GFP, treated with agrin for 10 days .

B) Quantification of peripheral nuclei in myofibers transfected with *GAPDH* siRNA (light blue fill) or *Amph2* ex11 siRNA (blue), GFP or GFP-VCA, and treated with agrin for 10 days. Error bars, s.e.m. p values from t-test (*GAPDH* siRNA vs *Amph2 ex11* siRNA).

C) Quantification of transversal triads in myofibers transfected with *GAPDH* siRNA (light blue fill) or *Amph2* ex11 siRNA (blue), GFP or GFP-VCA, and treated with agrin for 10 days. Error bars, s.e.m. p values from t-test (*GAPDH* siRNA vs *Amph2 ex11* siRNA).

D) Representative immunofluorescence images of myofibers transfected with *Amph2* ex3 siRNA and TdT (top) or TdT-VCA (bottom), treated with agrin for 10 days and stained for DHPR (red) and DAPI (blue).

E) Quantification of peripheral nuclei in myofibers transfected with *GAPDH* siRNA or *Amph2* siRNA, TdT or TdT-VCA, treated with agrin for 10 days. Error bars, s.e.m. p values from t-test (*GAPDH* siRNA vs *Amph2* siRNA).

F) Quantification of the percentage of transversal triads in myofibers transfected with *GAPDH* siRNA or *Amph2* siRNA, TdT or TdT-VCA, treated with agrin for 10 days. Error bars, s.e.m. p values from t-test (*GAPDH* siRNA vs *Amph2* siRNA).

Figure S5: Amph2 and N-WASP localization in mouse muscle

A) Representative immunofluorescence images of a transversal section of mouse muscle immunostained for DHPR (red) and N-WASP (green). Bar, 10 μm.

B) Left panels: representative immunofluorescence images of isolated mouse muscle fibers, immunostained for α-actinin (red) and N-WASP (green). Right panel: line-scan of indicated regions in the left panels showing average intensity of N-WASP (green) and α-actinin (red). Bar, 1 μm.

C) Left panels: representative immunofluorescence images of an isolated mouse muscle fibers, immunostained for DHPR (red) and N-WASP (green). Right panel: line-scan of indicated regions in the left panels showing average intensity of N-WASP (green) and DHPR (red) stainings, respectively. Bar, 1 μm.

D) Left panels: representative immunofluorescence images of an isolated mouse muscle fiber, immunostained for α-actinin (red) and Amph2 (green). Right panel: line-scan analysis of indicated regions in the left panel showing average intensity of Amph2 staining (green) and α-actinin (red) respectively. Bar, 1 μm.

E) Left panel: Representative immunofluorescence images of WT primary myofibers treated with agrin for 10 days and immunostained for DHPR (red), N-WASP (green) and DAPI (blue). Bar, 15 μm; Right panel: line-scan of indicated region in the left panel showing average intensity of N-WASP (green) and DHPR (red), respectively. Bar, 1 μm.

Figure S6: *in vivo* siRNA and plasmid DNA electroporation and fiber isolation

A) Representative images of isolated muscle fibers 10 days after electroporation of siRNA and TdT, showing negative (not expressing TdT) and positive (expressing TdT) myofibers . Left panel: TdT fluorescence. Right panel: Brightfield. Bar, 100 μm.
B) Representative immunofluorescence images of an isolated mouse muscle fibers, electroporated with *GAPDH* siRNA, *Amph2* siRNA or *N-WASP* siRNA, and immunostained for Amph2 (red) and N-WASP (green). Bar, 1 μm.
C) Representative image of isolated muscle fibers 10 days after electroporation of GFP-tagged vectors, showing negative (not expressing GFP) and positive (expressing GFP) myofibers . Left panel: GFP fluorescence. Right panel: Brightfield. Bar, 100 μm.
D) Representative immunofluorescence images of an isolated mouse muscle fibers, electroporated with GFP, GFP-Amph2-R154Q and GFP-Amph2-K573X, and immunostained for N-WASP. Bars, 1 μm.

Figure S7: α-actinin and F-actin organization in myofibers

A) Representative immunofluorescence images of an isolated mouse muscle fiber electroporated with *GAPDH* siRNA, *Amph*2 siRNA and *N-WASP* siRNA, immunostained for α-actinin (red) and F-Actin (green). Bars, 1 μm.

B) Representative immunofluorescence images of isolated mouse muscle fiber electroporated with CTRL GFP, GFP-AMPH2 R154Q and GFP-AMPH2K573X, respectively, and immunostained for α-actinin (red) and F-Actin (green). Bars, 1 μm.

Figure S8: N-WASP and Amph2 localization in muscle from healthy, XLCNM and ADCNM patients

A) Western blot analysis of control and ARCNM human biopsies homogenates after differential centrifugation, with N-WASP, Amph2, DHPR, Caveolin-3 (cav3) and α-Actinin antibodies.
B) Histogram with the quantification of (A), corresponding to the distribution (relative percentage of total protein) of N-WASP, Amph2, DHPR, cav3 and α-Actinin in different cellular fractions (1500g - myofibrils, 15000g -Organelle membranes and extrasarcomeric structures, 150000g - microsomes and macrocomplexes and cytosol). Error bars, s.e.m
C) Representative immunofluorescence images of transversal sections of human muscle from healthy donor (top) and ADCNM patient carrying DNM2 Q368K mutation (bottom), immunostained for DHPR (red), N-WASP (green) and DAPI (blue). Bars, 15 µm.
D) Representative immunofluorescence images of transversal sections of human muscle from healthy donor (CTRL, top) or XLCNM patient carrying R421insFIG mutation (bottom), immunostained for DHPR (red), N-WASP (green) and DAPI (blue). Bars, 15 µm.
E) Representative immunofluorescence images of transversal sections of human muscle from healthy donor (CTRL, top) or ADCNM patient carrying DNM2 Q368K mutation (bottom), immunostained for Amph2 (red), N-WASP (green) and DAPI (blue). Bars, 15 µm.
F) Representative immunofluorescence images of transversal sections of human muscle from healthy donor (CTRL, top) or XLCNM patient carrying R421insFIG (bottom), immunostained for Amph2 (red), N-WASP (green) and DAPI (blue). Bars, 15 µm.

Fig S9: mRNA and protein expression in muscle from healthy, XLCNM, ARCNM, ADCNM and DM1 patients

A) Relative mRNA expression of N-WASP, DHPR, Amph2, DNM2 and MTM1 in muscle biopsies from healthy (CTRL), XLCNM, ARCNM, ADCNM and DM1 patients assessed by real time PCR.

B) Western blot with Amph2, N-WASP, DHPR, DNM2, MTM1 and α-Actinin antibodies in CTRL, XLCNM, ADCNM, ARCNM from healthy or patient muscle biopsies carrying the indicated mutations. Levels may differ depending on patients.

C) Histograms of the relative protein expression of Amph2, N-WASP, DHPR, DNM2, MTM1 and DNM2 in CTRL, XLCNM, ADCNM, ARCNM from healthy or patient muscle biopsies showed in (B). Error bars, s.e.m. p values from t-test (patient versus ctrl conditions).

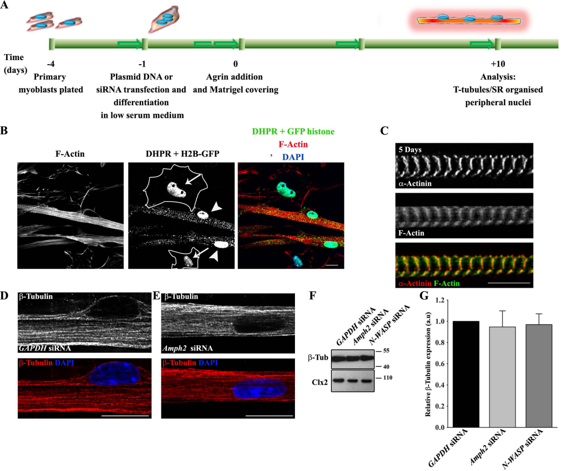
A
Time (days)
-4
Primary myoblasts plated
-1
Plasmid DNA or siRNA transfection and differentiation in low serum medium
0
Agrin addition and Matrigel covering
+10
Analysis: T-tubules/SR organised peripheral nuclei
B
F-Actin
DHPR + H2B-GFP
DHPR + GFP histone
F-Actin
DAPI
C
5 Days
α-Actinin
F-Actin
α-Actinin F-Actin
D
β-Tubulin
GAPDH siRNA
β-Tubulin DAPI
E
β-Tubulin
Amph2 siRNA
β-Tubulin DAPI
F
GAPDH siRNA
Amph2 siRNA
N-WASP siRNA
β-Tub
Clx2
55
40
110
G
Relative β-Tubulin expression (a.u)
1.2
1.0
0.8
0.6
0.4
0.2
0.0
GAPDH siRNA
Amph2 siRNA
N-WASP siRNA

Fig S1

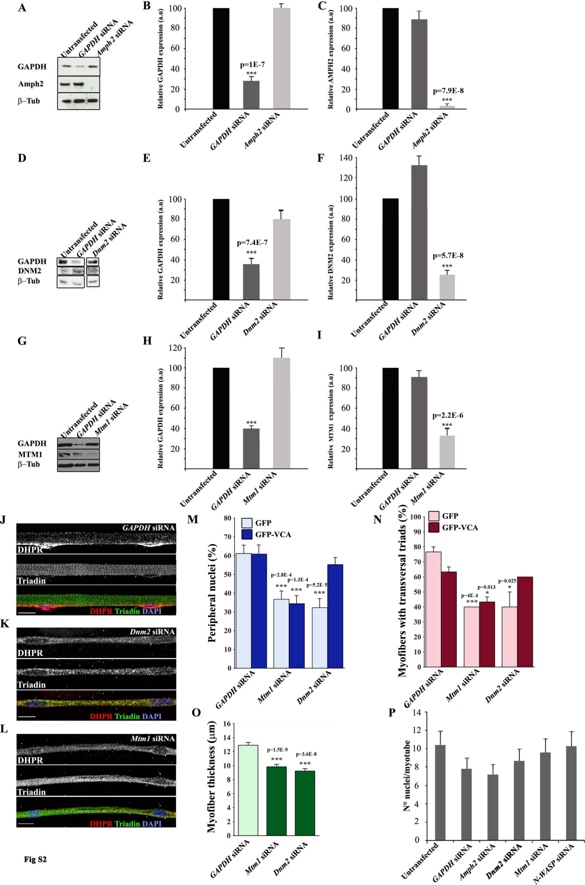
A
Untransfected
GAPDH siRNA
Amph2 siRNA
GAPDH
Amph2
β–Tub
B
Relative GAPDH expression (a.u)
p=1E-7
***
C
Relative AMPH2 expression (a.u)
p=7.9E-8
***
D
Dnm2 siRNA
DNM2
E
p=7.4E-7
F
Relative DNM2 expression (a.u)
p=5.7E-8
G
Mtm1 siRNA
MTM1
H
I
Relative MTM1 expression (a.u)
p=2.2E-6
J
GAPDH siRNA
DHPR
Triadin
DHPR Triadin DAPI
K
L
M
GFP
GFP-VCA
Peripheral nuclei (%)
p=2.8E-4
p=1.3E-4
p=5.2E-5
N
Myofibers with transversal triads (%)
p=4E-4
p=0.013
p=0.025
O
Myofiber thickness (µm)
p=1.5E-9
p=3.6E-8
P
N° nuclei/myotube
N-WASP siRNA

Fig S2

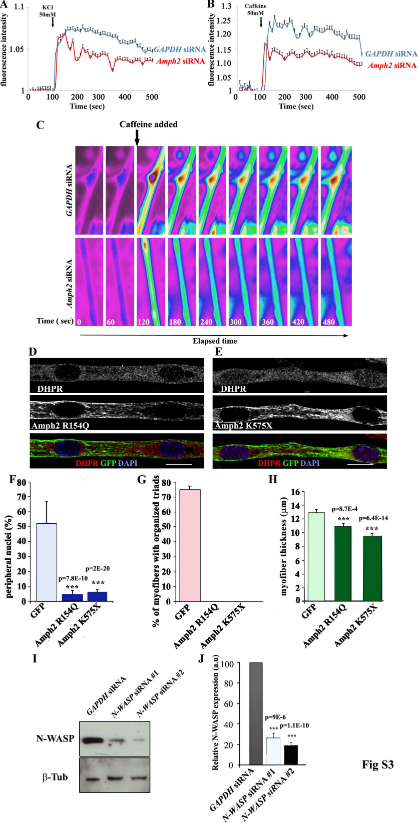

A
KCl 50mM
fluorescence intensity
GAPDH siRNA
Amph2 siRNA
Time (sec)
B
Caffeine 50mM
fluorescence intensity
GAPDH siRNA
Amph2 siRNA
Time (sec)
C
Caffeine added
GAPDH siRNA
Amph2 siRNA
Time (sec) 0 60 120 180 240 300 360 420 480
Elapsed time
D
DHPR
Amph2 R154Q
DHPR GFP DAPI
E
DHPR
Amph2 K575X
DHPR GFP DAPI
F
peripheral nuclei (%)
p=7.8E-10
p=2E-20
GFP
Amph2 R154Q
Amph2 K575X
G
% of myofibers with organized triads
GFP
Amph2 R154Q
Amph2 K575X
H
myofiber thickness (µm)
p=8.7E-4
p=6.4E-14
GFP
Amph2 R154Q
Amph2 K575X
I
GAPDH siRNA
N-WASP siRNA #1
N-WASP siRNA #2
N-WASP
β-Tub
J
Relative N-WASP expression (a.u)
p=9E-6
p=1.1E-10
GAPDH siRNA
N-WASP siRNA #1
N-WASP siRNA #2

Fig S3

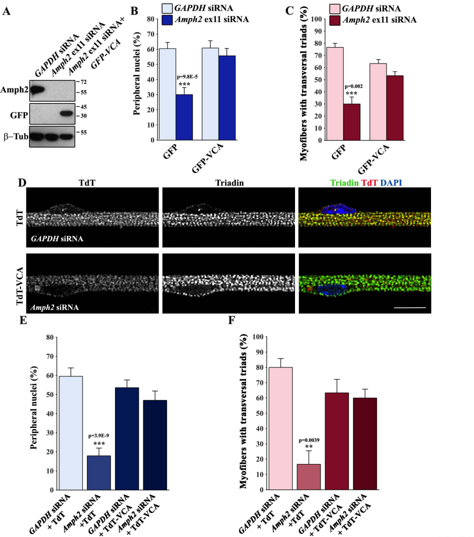
A
GAPDH siRNA
Amph2 ex11 siRNA
Amph2 ex11 siRNA+ GFP-VCA
Amph2
GFP
β–Tub
72
55
45
30
55
B
GAPDH siRNA
Amph2 ex11 siRNA
Peripheral nuclei (%)
p=9.8E-5
***
GFP
GFP-VCA
C
GAPDH siRNA
Amph2 ex11 siRNA
Myofibers with transversal triads (%)
p=0.002
***
GFP
GFP-VCA
D
TdT
Triadin
Triadin TdT DAPI
TdT
GAPDH siRNA
TdT-VCA
Amph2 siRNA
E
Peripheral nuclei (%)
p=3.9E-9
***
GAPDH siRNA + TdT
Amph2 siRNA +TdT
GAPDH siRNA + TdT-VCA
Amph2 siRNA + TdT-VCA
F
Myofibers with transversal triads (%)
p=0.0039
**
GAPDH siRNA + TdT
Amph2 siRNA +TdT
GAPDH siRNA + TdT-VCA
Amph2 siRNA + TdT-VCA

Fig S4

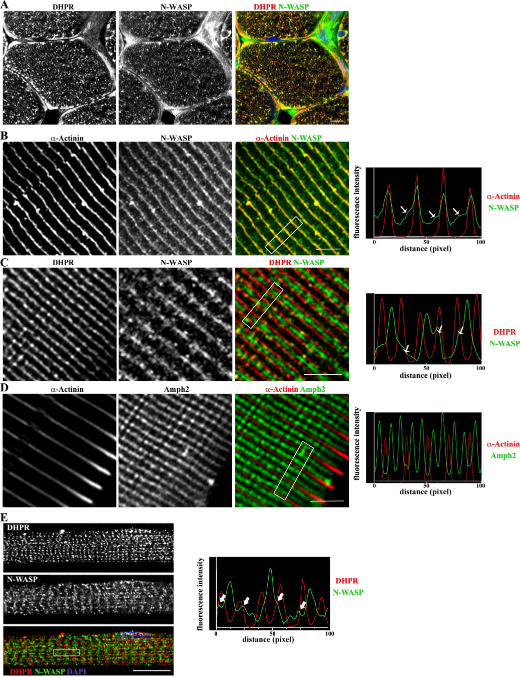

A
DHPR
N-WASP
DHPR N-WASP
B
α-Actinin
N-WASP
α-Actinin N-WASP
fluorescence intensity
distance (pixel)
0
50
100
α-Actinin
N-WASP
C
DHPR
N-WASP
DHPR N-WASP
distance (pixel)
0
50
100
DHPR
N-WASP
D
α-Actinin
Amph2
α-Actinin Amph2
fluorescence intensity
distance (pixel)
0
50
100
α-Actinin
Amph2
E
DHPR
N-WASP
DHPR N-WASP DAPI
fluorescence intensity
distance (pixel)
0
50
100
DHPR
N-WASP

Fig S5

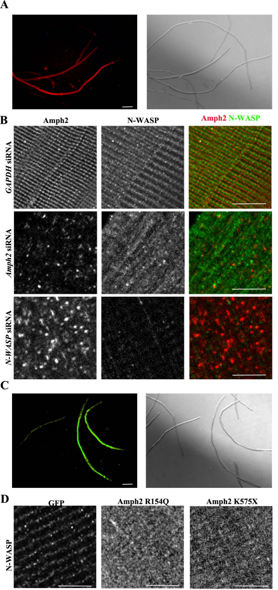

A
B
Amph2
N-WASP
Amph2 N-WASP
GAPDH siRNA
Amph2 siRNA
N-WASP siRNA
C
D
GFP
Amph2 R154Q
Amph2 K575X
N-WASP

fig S6

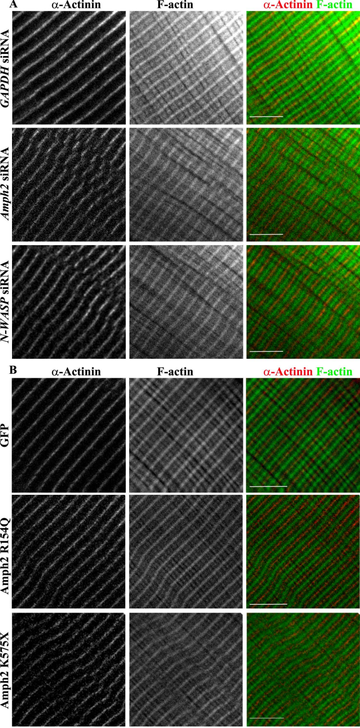
A
α-Actinin
F-actin
α-Actinin F-actin
GAPDH siRNA
Amph2 siRNA
N-WASP siRNA
B
α-Actinin
F-actin
α-Actinin F-actin
GFP
Amph2 R154Q
Amph2 K575X

Fig S7

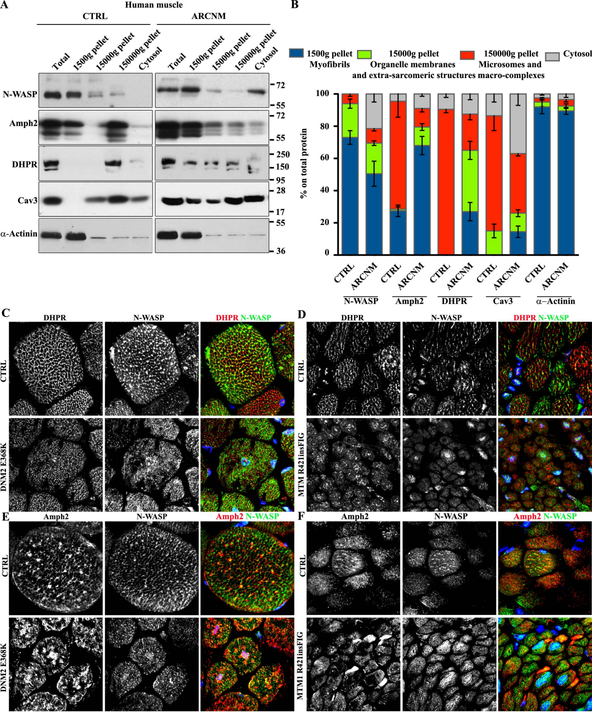
A
Human muscle
CTRL
ARCNM
Total
1500g pellet
15000g pellet
150000g pellet
Cytosol
N-WASP
Amph2
DHPR
Cav3
α-Actinin
72
55
72
55
250
150
95
28
17
55
36
B
1500g pellet
Myofibrils
15000g pellet
Organelle membranes
and extra-sarcomeric structures
150000g pellet
Microsomes and
macro-complexes
Cytosol
% on total protein
100
80
60
40
20
0
CTRL
ARCNM
N-WASP
Amph2
DHPR
Cav3
α–Actinin
C
DHPR
N-WASP
DHPR N-WASP
CTRL
DNM2 E368K
D
DHPR
N-WASP
DHPR N-WASP
CTRL
MTM R421insFIG
E
Amph2
N-WASP
Amph2 N-WASP
CTRL
DNM2 E368K
F
Amph2
N-WASP
Amph2 N-WASP
CTRL
MTM1 R421insFIG

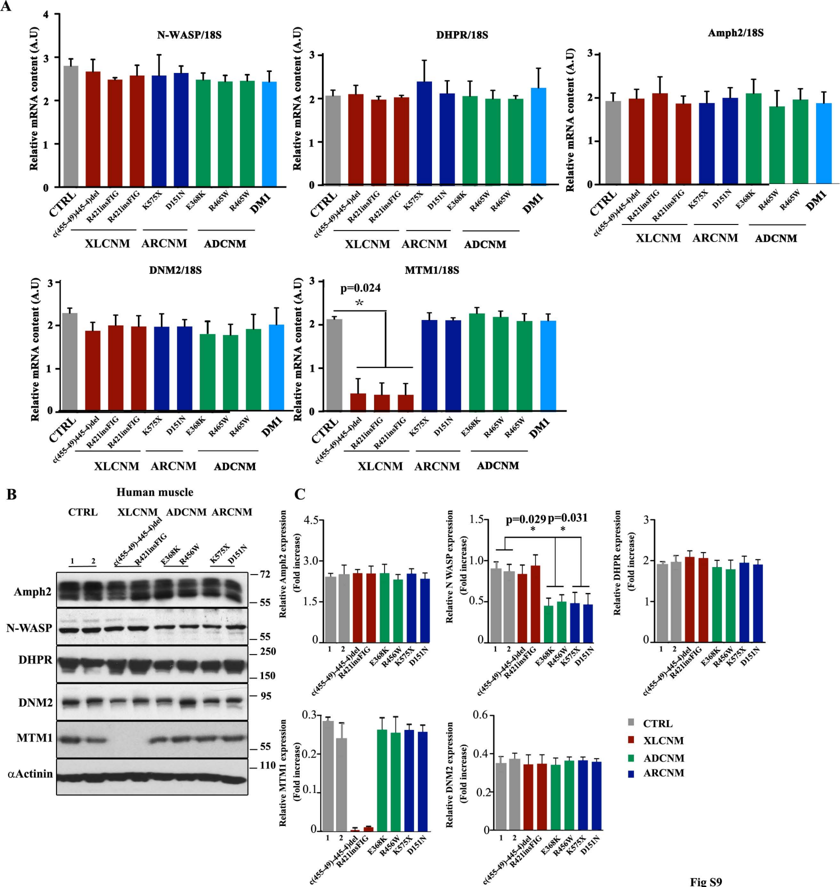

A
N-WASP/18S
DHPR/18S
Amph2/18S
DNM2/18S
MTM1/18S
p=0.024
Relative mRNA content (A.U)
CTRL
c(455-49)445-4)del
R421insFIG
K575X
D151N
E368K
R465W
DM1
XLCNM
ARCNM
ADCNM
B
Human muscle
CTRL
XLCNM
ADCNM
ARCNM
Amph2
N-WASP
DHPR
DNM2
MTM1
αActinin
C
Relative Amph2 expression (Fold increase)
Relative N WASP expression (Fold increase)
p=0.029
p=0.031
Relative DHPR expression (Fold increase)
Relative MTM1 expression (Fold increase)
Relative DNM2 expression (Fold increase)
R456W
CTRL
XLCNM
ADCNM
ARCNM

Fig S9
